# Pan-genome analysis reveals structural variation- associated expression and evolutionary diversity of the *ZmCYP450* gene family in maize

**DOI:** 10.3389/fpls.2026.1874015

**Published:** 2026-07-15

**Authors:** Weijian Qi, Jingcheng Wang, Jianzhong Chang, Huahu Bu, Jianhong Xiao, Ning Zhang, Zhiqiang Ren

**Affiliations:** 1 College of Agronomy, Shanxi Agricultural University, Jinzhong, China; 2 Key Laboratory of Organic Dry Farming, Ministry of Agriculture and Rural Affairs, Shanxi Agricultural University Key Laboratory of Organic Dry Farming in Shanxi Province Country, Taiyuan, China

**Keywords:** copy number variation, cytochrome P450 (CYP450), maize, pan-genome, structural variation, tissue-specific expression

## Abstract

The plant cytochrome P450 (CYP450) superfamily plays a key role in metabolic diversity and environmental adaptation; however, systematic analyses of its intraspecific structural variation, copy number dynamics, and evolutionary mechanisms remain limited. Using 27 high-quality maize reference genomes, we performed a pan-genomic analysis of the ZmCYP450 family, identifying 282 orthogroups (OGs) and 7,282 genes. The family exhibits a pattern of predominantly conserved genes with localized expansions and open pan-genome properties. PAV and CNV analyses revealed extensive gene deletions and copy number fluctuations outside core OGs, reflecting substantial intraspecific structural diversity. Analysis of duplication types and local colinearity suggested that proximal and dispersed duplications are the primary contributors to drive family expansion. Ka/Ks analysis indicated that most OGs are under purifying selection, while a subset shows evidence of positive selection. Further integration of structural variation and transcriptomic data suggested that SVs may affect gene function through mechanisms such as gene deletion, protein truncation, and remodeling of regulatory elements, suggesting a dual role of potential loss of function and expression modulation. Despite a relatively stable overall copy number, structural variant categories (‘Typical’, ‘Atypical’, and ‘Missing’) are widespread, and expression levels do not always correlate with copy number, suggesting a complex regulatory patterns. Tissue-specificity analysis revealed a large number of highly specific *ZmCYP450* genes involved in secondary metabolism and environmental responses, with distinct expression profiles across different genetic backgrounds. This study provides a comprehensive pan-genomic view of structural variation, evolutionary patterns, and expression regulation in the ZmCYP450 family, offering a foundation for future functional studies and maize trait improvement.

## 1 Introduction

Cytochrome P450 enzymes are a superfamily of heme proteins with multifunctional oxidase activity, widely distributed in animals, plants, microorganisms, and fungi (Mao et al., 2013). These enzymes are typically localized to membrane structures such as the endoplasmic reticulum, mitochondria, plastids, and the Golgi apparatus. Acting as terminal oxidases, they participate in various biosynthetic reactions, play a broad role in numerous metabolic processes within plants, and exhibit significant diversity in substrate recognition and catalytic functions. These enzymes were first discovered in cotton in 1969 (Frear et al., 1969) and have since been shown to play key roles in secondary metabolism, hormone regulation, and environmental responses.

In recent years, studies have shown that the CYP450 family has undergone significant expansion in plants. Although its protein structure is highly conserved, different members show great differences in function, thus widely participating in plant primary and secondary metabolic processes. Studies have confirmed that plant CYP450 plays a key role in a variety of biological processes, including the synthesis of secondary metabolites (such as phenylpropanoids, terpenoids and alkaloids), the metabolic regulation of plant hormones (such as abscisic acid, gibberellin and brassinolide), and the response to abiotic stresses such as drought, salinity, and low temperature stress (Schuler and Werck-Reichhart, 2003).Therefore, it is known as the ‘universal catalyst’ (Schuler and Werck-Reichhart, 2003). In Catharanthus roseus, the expression pattern of *CYP72A1* is highly consistent with the expression of TDC and STR in the indole alkaloid synthesis pathway. Heterologous expression experiments have confirmed that *CYP72A1* can catalyze the oxidative cleavage of loganin to produce silymarin, which has become an important node in the regulation of secondary metabolic production (Irmler et al., 2000). Similarly, the overexpression of *CYP78A5* can cause stem bending and abnormal flower development, which may be related to the regulation of lignin biosynthesis through phenylpropanoid pathway, thus affecting cell wall characteristics and cell growth (Zondlo and Irish, 1999). In addition, the functional study of the *CYP709B* subfamily showed that *CYP709B3* showed a significant response to salt stress and ABA, suggesting that it played a key role in the process of stress adaptation (Mao et al., 2013). Therefore, the CYP450 family plays an important role in plant growth and development and environmental adaptation.

With the development of molecular biology and omics technology, significant progress has been made in the systematic identification and functional study of CYP450 family in a variety of plants. In the study of non-model plants, researchers have gradually revealed the core role of CYP450 in metabolic networks by integrating genomic and metabolomic data. TingYang et al. identified 180 *SmCYP* members in the eggplant genome, and verified the significant tissue expression specificity of the family by qRT-PCR. At the same time, combined with metabolomics data, they confirmed the important role of *SmCYP73A1*, *SmCYP75A* and *SmCYP98A1* in flavonoid synthesis (Yang et al., 2024). Similarly, Chen et al. analyzed the transcriptome of 248 *CYP450* genes in the chrysanthemum genome. The results showed that most of the members showed specific expression in leaves, stems and roots, and confirmed that 36 genes such as *CiCYP706–5* and *CiCYP71–50* were critical in the biosynthesis of volatile organic compounds (Chen et al., 2025). At the technical level, Xu et al. first used microarray technology to systematically evaluate 273 family members of thale cress (*Arabidopsis thaliana* (L.) Heynh.). The optimized probe design reduced the cross-interference between homologous genes and significantly improved the accuracy of P450 gene expression detection (Xu et al., 2001). In addition, comparative genomics and systems biology methods further revealed the synergistic regulatory network of CYP450: Li et al. constructed a gene co-expression network (WGCNA) and found that members co-expressed with lipoxygenase (LOX) and 12-oxo plant diene acid reductase (OPR) were considered to be involved in jasmonic acid (JA) -mediated stress response; the genes co-expressed with core components such as PAL, CCR and COMT are closely related to the lignin deposition process (Li and Wei, 2020). These multidimensional studies not only depict the functional panorama of the CYP450 family, but also provide an important theoretical basis for understanding the functional differentiation and metabolic evolution mechanism of the family in the long-term evolution of plants.

Maize (*Zea mays* L.) is not only an important cereal crop, but also a model plant species for genetics, cytology, genomics and molecular research. It has a complex genomic structure and rich genetic diversity (Li et al., 2025). Although the existing studies have carried out preliminary identification and functional prediction of the ZmCYP450 family (Chakraborty et al., 2023), they often focus on transcriptome analysis of single tissues or specific varieties, and lack systematic comparison across varieties, which to some extent limits our in-depth understanding of the evolutionary pattern of the ZmCYP450 family and its relationship with phenotypic variation and environmental adaptability of maize. Especially in the context of multiple genetic backgrounds, differences in expression regulation and gene structure of *CYP450* genes may be an important factor in promoting their functional differentiation, but related research in this area is still relatively scarce. In addition, as a key genetic basis affecting gene function and expression, structural variations (SVs) is widely present in the maize genome (Hufford et al., 2021), but its distribution characteristics and functional significance in the ZmCYP450 family are still lack of systematic analysis. Therefore, the integration of genomic structure information and transcriptome information on the basis of multiple varieties, through a comprehensive analysis of the ZmCYP450 family, will help to reveal how the family maintains a dynamic balance between functional conservation and expression diversity during evolution, and further clarify its potential mechanism in maize growth and development and stress response.

Against this backdrop, this study will address the following questions: In complex genomic contexts and diverse genetic environments, how does the ZmCYP450 family in maize maintain the stability of its core functions while achieving diversity in gene structure and expression patterns? Furthermore, do the expression differences and structural variations of *ZmCYP450* genes among different maize varieties reveal potential mechanisms underlying their adaptive evolution?

Accordingly, this study drew upon cutting-edge paradigms in the field of plant pan-genomics (Zhao et al., 2026). Based on 27 high-quality maize reference genomes, we systematically integrated research methods—including orthogroups identification, evolutionary and selective pressure analysis, phylogenetic analysis, and large-scale structural variations (SVs) scanning—at the pan-genomic level for the first time, comprehensively elucidating the evolutionary dynamics and functional differentiation characteristics of the ZmCYP450 family. Building on this foundation, we combined multi-tissue expression data with expression differences across varieties to systematically analyze the expression diversity of this family and explore the potential impact of structural variations on its function. This study not only provides a new theoretical framework for elucidating the molecular mechanisms underlying the ZmCYP450 family’s role in metabolic regulation and stress response in maize but also offers valuable candidate genes and molecular targets for maize trait improvement.

## 2 Materials and methods

### 2.1 Genome data acquisition and *ZmCYP450* gene identification

To systematically identify ZmCYP450 family genes across the maize pan−genome, the genome−wide protein sequence files of 27 representative maize inbred lines were retrieved from the MaizeGDB (https://maizegdb.org/) database (**Supplementary Table 1**). Genome-wide identification was performed using a hidden Markov model (HMM) search strategy based on structural domain characteristics. First, the HMM profile corresponding to the cytochrome P450 core domain (PF00067) was downloaded from the Pfam database (https://www.ebi.ac.uk/interpro/entry/pfam/PF00067/). Subsequently, the hmmsearch program within the HMMER software package (v3.3.2) was deployed in an Ubuntu (v22.04.5) environment to perform a large-scale screening against the local protein databases of the 27 maize varieties, with the statistical *E*-value threshold strictly set at 1×10^-5^. Given the prevalence of multiple transcripts generated by alternative splicing within the maize genome, a rigorous deduplication workflow was implemented to eliminate redundancy in downstream analyses. All candidate sequences were mapped back to their respective genomic loci (gene locus); for multiple isoforms derived from a single locus, only the longest protein sequence (longest isoform) was retained as the unique representative for that gene. Finally, the NCBI Conserved Domain Database (https://www.ncbi.nlm.nih.gov/cdd/) was employed to evaluate and verify the domain integrity of all primarily screened candidate proteins. To comprehensively preserve the entire spectrum of genetic evolutionary variations within the maize pan-genome—including unstable fragments undergoing rapid evolution or pseudogenization—an inclusive retention strategy was adopted during this primary identification stage. Specifically, sequences were retained as long as they passed the stringent HMMER statistical threshold (*E ≤* 1×10^-5^) and harbored the characteristic conserved domains associated with cytochrome P450. Consequently, a comprehensive pan-genomic matrix of candidate *ZmCYP450* genes across the 27 maize varieties was defined and compiled (**Supplementary Table 2**).

### 2.2 Orthogroup identification and pan-genomic characterization

In order to study the homologous relationship between 27 maize varieties and construct a pan-genome, we used OrthoFinder (v2.5.5) for orthogroups analysis. In the Ubuntu (v22.04.5) environment, the DIAMOND algorithm was called to perform all-versus-all protein sequence alignment, and the MCL algorithm was used to cluster the genes into orthogroups based on sequence similarity. After the clustering is completed, the gene counting matrix of each variety is extracted from the output file. According to the distribution frequency of each orthogroup in 27 genomes, the classification criteria of Tong et al (Tong et al., 2025). These 282 OGs were divided into four evolutionary components: Core OGs (present in all 27 genomes), Soft-core OGs (present in ≥ 90% of the 27 genomes), Dispensable OGs (present in 2% -90% of the 27 genomes) and Private OGs (present only one of the 27 genomes). Finally, the copy number matrix is reshaped and the multidimensional bubble diagram is visualized by using tidyverse and ggnewscale software packages in R (v4.5.3).

### 2.3 Phylogenetic tree analysis of *ZmCYP450* gene family

For phylogenetic analysis, a dataset comprising 282 representative maize P450 protein sequences (one extracted as the representative from each of the 282 orthogroups) and 272 *Arabidopsis* reference sequences was utilized. To determine the representative sequence for each orthogroup, the sequence with the highest structural integrity among those with ≥95% integrity in the conserved core region was selected. Multiple sequence alignment of the combined sequences was performed using MAFFT (v7.490), followed by automated trimming with TrimAl (v1.4.1) under the ‘-automated1’ mode to remove low-quality alignment regions and retain the conserved core columns. The reference sequences for *Arabidopsis* P450 proteins were retrieved from The Cytochrome P450 Homepage (https://www.p450.kvl.dk/p450.shtml). A maximum-likelihood phylogenetic tree was subsequently constructed using IQ-TREE2 (v2.1.2), with the best-fit amino acid substitution model automatically selected by the built-in ModelFinder program. Branch support was evaluated using ultra-fast bootstrap (UFBoot) analysis with 1,000 replicates.

Concurrently, a local BLASTP (v2.16.0) search was executed in an Ubuntu (v22.04.5) environment, using the *Arabidopsis* P450 protein dataset as the reference database to query the candidate maize sequences with an *E*-value threshold of ≤ 1e^-5^. Subsequently, the raw BLAST output was filtered and processed using the Tidyverse package in R (v4.5.3). Only the top-hit *Arabidopsis* match (the alignment with the highest Bit-score) for each query gene was retained. The corresponding CYP family and clan information were then extracted from these top hits to serve as an auxiliary validation for functional annotation and subfamily categorization.

### 2.4 Analysis of presence-absence variation and copy number variation in *ZmCYP450* genes

Based on the gene copy number matrix (**Supplementary Table 3**) obtained through clustering using OrthoFinder (v2.5.5), we utilized the ComplexHeatmap package and its dependent package Circlize in R (v4.5.3) to generate PAV and CNV panoramic heatmaps for 282 ZmCYP450 family OGs. The PAV heatmap represents presence/absence status through binarization, while the CNV heatmap displays copy number variations using a continuous color gradient, ultimately visualizing the ZmCYP450 family heatmaps across 27 varieties. To validate the PAV and CNV patterns inferred from orthogroup occupancy, two representative PAV orthogroups and two representative CNV orthogroups were selected. For PAV loci, structural variation (SV) datasets generated from whole-genome comparisons were used to examine whether deletion events overlapped the target gene regions. For CNV loci, gene coordinates and neighboring gene information were extracted from genome annotation (GFF3) files, and local microsynteny relationships were visualized in R (v4.5.3) using the ggplot2 and gggenes packages. Sequence similarity among duplicated copies was further evaluated based on pairwise protein identity. These analyses were used to determine whether the inferred PAV and CNV signals corresponded to genuine genomic structural variations rather than annotation artifacts.

### 2.5 Gene replication, collinearity and evolutionary analysis

In this study, the duplication patterns of *ZmCYP450* genes in 27 maize varieties were systematically classified. Using B73RefGen_v5 as a reference, homology was identified and GFF3 coordinates were standardized via BLASTp (v2.16.0) with an *E*−value ≤ 10^-5^ and identity ≥ 80%. Based on their spatial distribution, the 7,282 genes were divided into five duplication types: WGD, TD, PD, TRD, and DSD. Collinearity among varieties was visualized using the ggplot2 package. Finally, the 282 orthogroups of the ZmCYP450 family were categorized into three evolutionary classes based on occupancy: Core, Soft−core, and Dispensable.

### 2.6 Ka/Ks calculation

We extracted the corresponding protein sequences and coding sequences (CDSs) from homologous genomes. We first performed multiple sequence alignment on the protein sequences using MAFFT (v7.490), and then mapped the protein alignment results back to the CDS sequences using PAL2NAL (v14.1) to construct codon-level nucleotide alignments. For each member within an OG, we constructed all-vs-all pairwise combinations. Based on this, we used the YN00 model in KaKs Calculator (v3.0) to calculate the non-synonymous substitution rate (Ka), the synonymous substitution rate (Ks), and their ratio (Ka/Ks) for each pair of homologous genes. For each OG, the Ka/Ks values of all valid homologous gene pairs within the group were aggregated, and the median was used as the representative indicator of selective pressure for that group to mitigate the influence of extreme Ka/Ks ratios on the results. Finally, in the R (v4.5.3) environment, the Ka/Ks distributions of each OG were visualized using ggplot2 and ggridges, and ridge plots were generated to illustrate differences in selective pressure among different groups.

### 2.7 Structural variation and gene expression and structural correlation analysis

Using MUMmer (v4.0.0beta2), the genomes of 26 maize inbred lines were individually aligned to the B73RefGen_v5 reference genome to perform whole-genome comparisons. Structural variants (SVs) were identified based on regions exhibiting alignment discrepancies, and only insertions (INS) and deletions (DEL) with lengths ≥50 bp were retained for downstream analyses, with the requirement that the aligned blocks have an identity ≥80%. Using genomic coordinates and gene annotation files, SVs overlapping the coding sequences (CDS) or the 2-kb upstream promoter regions of *ZmCYP450* genes were extracted. To minimize potential false positives arising from genome assembly differences, only SVs located within annotated gene regions and clearly identifiable from the whole-genome alignments were retained. The resulting SV dataset was subsequently used for gene structure comparisons, gene expression analyses, protein sequence evaluations, and cis-regulatory element analyses. At the expression level, combining RNA-seq transcriptomic data from five tissues, samples were divided into two groups based on SV status: ‘With SV’ and ‘Without SV’. Here, ‘With SV’ refers to genes with structural variations in the target gene region, while ‘Without SV’ refers to genes in which no corresponding SVs were detected. TPM expression levels were analyzed in the R (v4.5.3) environment, and the Wilcoxon rank-sum test was used to compare expression level differences between the two groups to assess the impact of structural variants on gene expression. At the protein structural level, protein sequences of candidate genes were extracted from B73 and corresponding mutant lines, and sequence alignment analysis was performed to evaluate the potential impact of structural variants on protein integrity. At the regulatory level, PlantCARE (https://bioinformatics.psb.ugent.be/webtools/plantcare/html/) was used to predict cis-acting elements in the promoter region, analyze the gain and loss of regulatory elements before and after SVs, and visualize the distribution and sequence characteristics of regulatory elements using ggplot2 and ggseqlogo in the R (v4.5.3) environment.

### 2.8 Identification and expression correlation analysis of typical and atypical genes

First, protein sequences from the 27 maize varieties were scanned for conserved CYP450 motifs using custom scripts implemented in R (v4.5.3). Based on protein length and the integrity of the characteristic CYP450 motifs, particularly the highly conserved heme-binding motif, ZmCYP450 family members were classified into three categories. ‘Typical’ genes were defined as proteins retaining a complete CYP450 domain architecture and an intact heme-binding motif. ‘Atypical’ genes were defined as proteins exhibiting structural truncation or the loss of one or more key CYP450 motifs/domains, including partial disruption of the heme-binding region. ‘Missing’ genes represented gene-loss events in which the corresponding orthologous CYP450 member was absent from a given genome and no recognizable CYP450 structural features could be detected. To evaluate the contribution of gene structural integrity to population-level expression diversity, TPM expression matrices from five representative tissues across 27 maize inbred lines were collected. For each accession, the numbers of typical and atypical genes were quantified separately, and Pearson correlation analysis was performed to examine the relationship between Typical genes and the total expression level of the corresponding ZmCYP450 family. Tissue-specific patterns were visualized using the faceting function implemented in the ggplot2 package of R (v4.5.3).

### 2.9 RNA-seq expression analysis

Raw RNA−seq data were obtained from EMBL−EBI (https://www.ebi.ac.uk) and from the NCBI SRA database (http://www.ncbi.nlm.nih.gov/sra). The accession numbers for the each RNA−seq datasets used in this study are listed in **Supplementary Table 4**. Specifically, we analyzed five core tissues (leaf, shoot, root, tassel, and ear) across 27 maize varieties. In this study, we first identified ZmCYP450 orthogroups with detectable expression in at least one tissue from 27 maize varieties. The expression matrices for all tissues were standardized and processed uniformly, retaining only ZmCYP450 OGs with detectable expression (TPM > 0) in at least one tissue and excluding genes with no expression in any tissue. Within each orthogroup, the gene with the highest τ value was selected as the representative gene for that OG to reflect the tissue-specific expression characteristics of that gene family. The calculation formula is as follows (Kryuchkova-Mostacci and Robinson-Rechavi, 2017):


$$\tau =\frac{\sum_{i}^{N}\left(1-{\hat{x}}_{i}\right)}{N-1},{\hat{x}}_{i}=\frac{T_{i}}{max\left(T\right)}$$

Where N is the number of tissues, *T_i_* represents the expression level (TPM) of a gene in tissue i, and *max(T)* is the maximum expression value of that gene across all tissues. *x_i_* is the normalized expression value of tissue *i*, scaled to the range [0,1]. Building on previous research (Lüleci and Yılmaz, 2022), we define thresholds based on the overall distribution of τ values across genes and classify them according to quantiles based on this distribution. To assess the expression plasticity of genes from different functional categories within the population, we calculated the coefficient of variation (CV = SD/Mean) of gene expression across the 27 varieties within each τ class, sorted the genes by CV value, and selected the top 7 orthogroups based on the CV of their representative gene from each class as representative genes to illustrate their expression variability characteristics at the population level. Furthermore, we employed an R script to specifically identify a class of orthogroups that contain multiple paralogous loci within a single maize genome. For these orthogroups, transcriptome expression matrices from different tissues and developmental stages were extracted and aligned to characterize the expression profiles of individual paralogous genes across various tissues.

## 3 Results

### 3.1 Composition and structural diversity of the ZmCYP450 family from a pan-genomic perspective

#### 3.1.1 Identification and compositional characteristics of the ZmCYP450 family in the maize pan-genome

**Figure 1:**
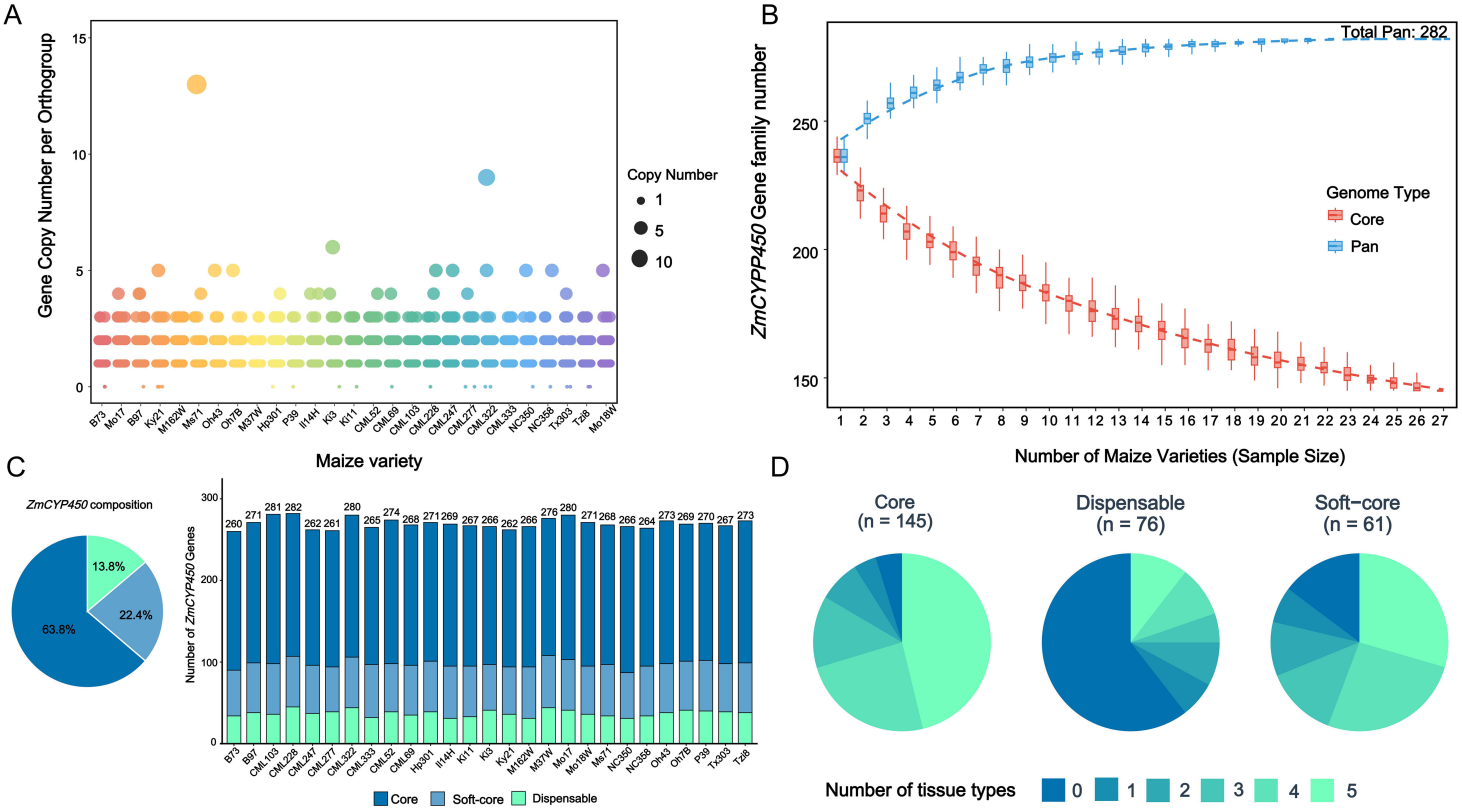
Pan-genomic characterization of the ZmCYP450 orthogroups. **(A)** Landscape of *ZmCYP450* gene family expansion across the maize pan-genome. Each bubble represents an orthogroup, with positions arranged by maize variety. Bubble size corresponds to the gene copy number; larger bubbles indicate a higher copy number for the OG in that specific variety. **(B)** Pan-genome and core-genome accumulation curves. The blue and red curves illustrate the expansion trend of the pan-genome and the changes in the number of core OGs, respectively, as the number of sampled genomes increases. **(C)** Proportional distribution of core, soft-core, and dispensable OGs within the ZmCYP450 family, and their distribution across the 27 maize varieties; different colors represent distinct OG categories. **(D)** Tissue-specific expression profiles for each OG category. The 5 tissues analyzed include ear, leaf, root, shoot, and tassel.

To comprehensively reflect the extensive genetic diversity within maize (*Zea mays* L.), this study analyzed a total of 27 high-quality, chromosome-level reference genomes. These materials represent multiple dimensions of genetic variation, covering major subpopulations such as stiff-stalk, non-stiff-stalk, and various tropical/temperate lines, as well as a wide range of market classes including yellow/white dent corn, flint corn, popcorn, and sweet corn. Notably, these materials exhibit significant variation in environmental stress tolerance, disease resistance, and key agronomic traits such as flowering time and plant architecture. Although some classical genomes are widely used in maize research, to ensure high comparability and accuracy of the whole-genome analyses, we preferentially selected the 27 high-quality genomes released on MaizeGDB (https://maizegdb.org/), thereby avoiding errors arising from technical annotation differences. Consequently, these 27 genomes, as a highly standardized dataset, are sufficient to precisely represent the genetic diversity and functional variation within the maize species (detailed classification and source information are provided in **Supplementary Table 1**. Using OrthoFinder (v2.5.5), we then identified orthogroups (OGs) of the *ZmCYP450* gene family from the whole-genome sequences of these 27 varieties to systematically analyze their genome-wide distribution characteristics, and counted the gene copy number of each OG in each variety, and finally visualized in the form of bubble chart (**Figure 1A**). In this study, we identified 282 OGs and 7282 *ZmCYP450* genes (**Supplementary Table 2**). Most of the OGs were concentrated in the range of 1–5 copies in different varieties, but showed significant variety-specific expansion in varieties such as Ms71 and CML322, with more than 5 or even more than 10 copies. On the whole, the OGs of the family showed a pattern of overall conservatism and local expansion. The pan-genome accumulation curve showed that with the increase of the number of genomes, the scale of the pan-genome gradually expanded, from the rapid growth in the early stage to the gradual slowdown in the growth rate in the later stage, and did not reach full saturation. In contrast, the number of core OGs gradually decreased with the increase of samples (**Figure 1B**). Furthermore, we classified 282 OGs, of which 145 were core OGs, accounting for the highest proportion in the ZmCYP450 family (63.8% of the total genes), 61 were soft-core OGs (22.4% of the total genes), and 76 were dispensable OGs, accounting for the lowest proportion in the family (13.8% of the total genes). Private OGs were not detected. All 27 maize varieties contained three types of ZmCYP450 OGs, and the number of genes contained in each variety was 260-282 (**Figure 1C**). Most of the genes in the core OGs that constitute the main body of the family have transcriptional activity in 1–5 tissues, representing their maintenance of basal metabolic stability (Thorrez et al., 2011). Genes belonging to dispensable OGs showed a significant tissue-specific expression pattern, and their expression was limited to specific organs or developmental stages; soft-core OGs are between the two, with a certain degree of conservatism (**Figure 1D**).

#### 3.1.2 Presence-absence variants and copy number dynamics in the ZmCYP450 family

**Figure 2:**
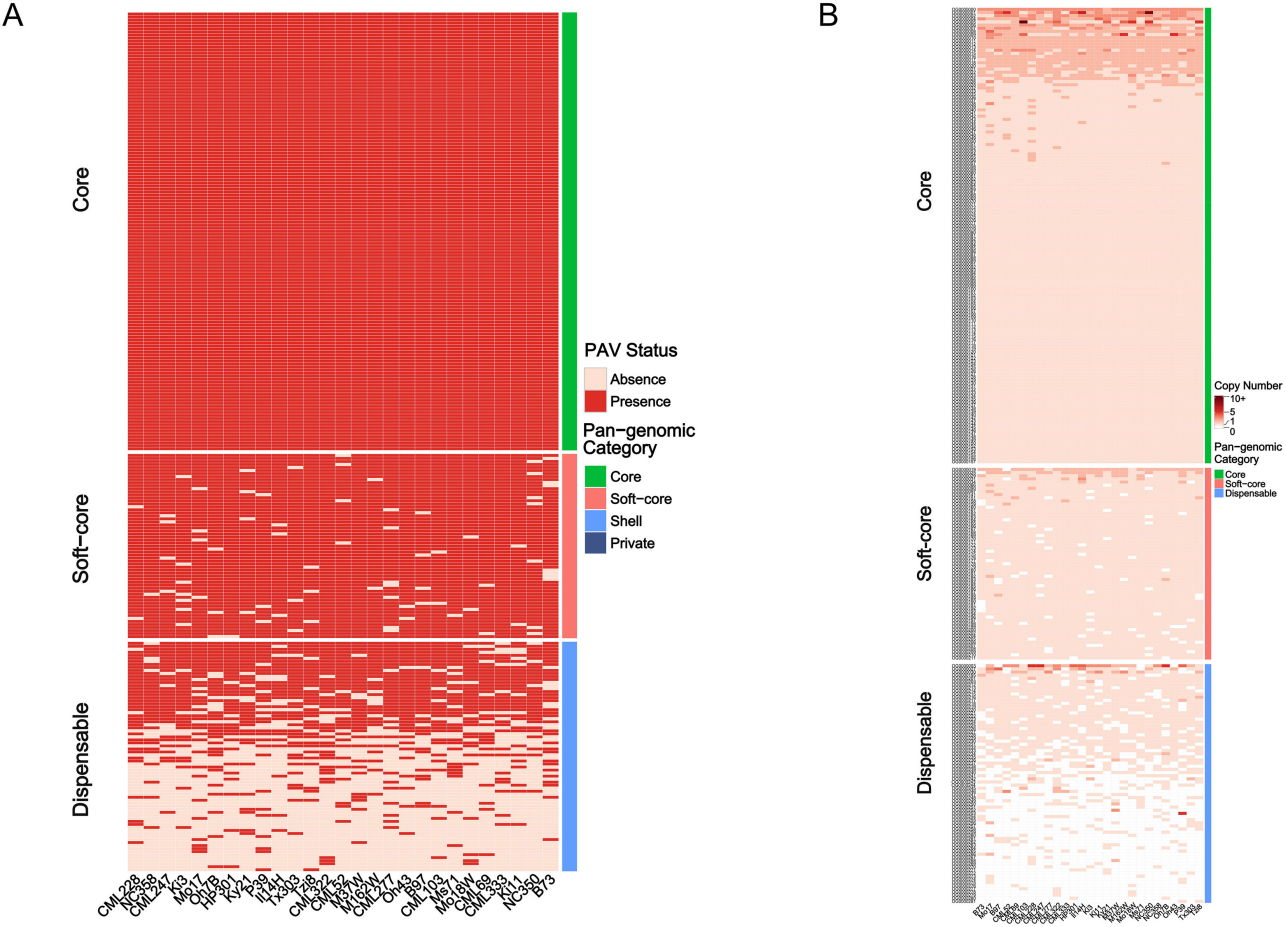
PAV and CNV analysis of the ZmCYP450 orthogroups. **(A)** The heatmap shows the presence and absence of the 282 ZmCYP450 orthogroups across 27 maize varieties. This family was classified into core OGs, soft-core OGs, and dispensable OGs. **(B)** CNV heatmap of the ZmCYP450 orthogroups in the maize genome. This figure illustrates the copy number variation (CNV) patterns of ZmCYP450 OGs across 27 maize varieties; darker regions indicate higher copy numbers, while lighter regions indicate lower copy numbers.

We constructed a visual heat map of PAV (presence/absence variation) and CNV (copy number variation) based on 282 OGs of 27 maize varieties. It can be observed from the PAV heat map that except for core OGs, CML322, CML333 and M37W did not show gene deletion in soft-core OGs, reflecting their high genomic integrity (**Figure 2A**). In dispensable OGs, there is a high proportion of deletions in all varieties. This significant difference between varieties reflects the variability of the *ZmCYP450* gene family, and these deletions may lead to differences in genome content (Della Coletta et al., 2021).

Analysis of the pan-genomic matrix revealed that member genes of the ZmCYP450 superfamily were distributed across 282 orthogroups, with the number of occupied OGs per genome fluctuating between 260 and 282 (**Figure 2B**). In terms of cumulative physical gene copies, the total size of the ZmCYP450 repertoire exhibited substantial variation among the 27 lines. Variety CML228 harbored the most expanded lineage with a total of 282 gene copies, indicating a significant lineage-specific expansion. In contrast, the reference line B73 possessed a relatively contracted genome architecture, retaining only 260 physical gene copies, and the difference in copy number between the two was about 8%. Combined with color gradient analysis, most core OGs maintained a low copy state in all varieties, and their CNV patterns also showed a high degree of consistency. Of course, there are also some OGs (such as OG0000000 ~ OG0000005) that maintain high copies in most or even all varieties. Compared with the stable performance of core OGs, dispensable OGs showed the most severe copy number fluctuations, such as the large-scale deletions of OG0000269, OG0000270, OG0000271 and other groups in most varieties, reflecting strong variety specificity. In contrast, groups such as OG0000003 and OG0000253 showed high copy status in specific varieties such as CML247, CML228, and P39. However, the soft-core OGs as an intermediate transition category did not show significant copy number fluctuations as a whole, with only a small number of deletions.

**Figure 3:**
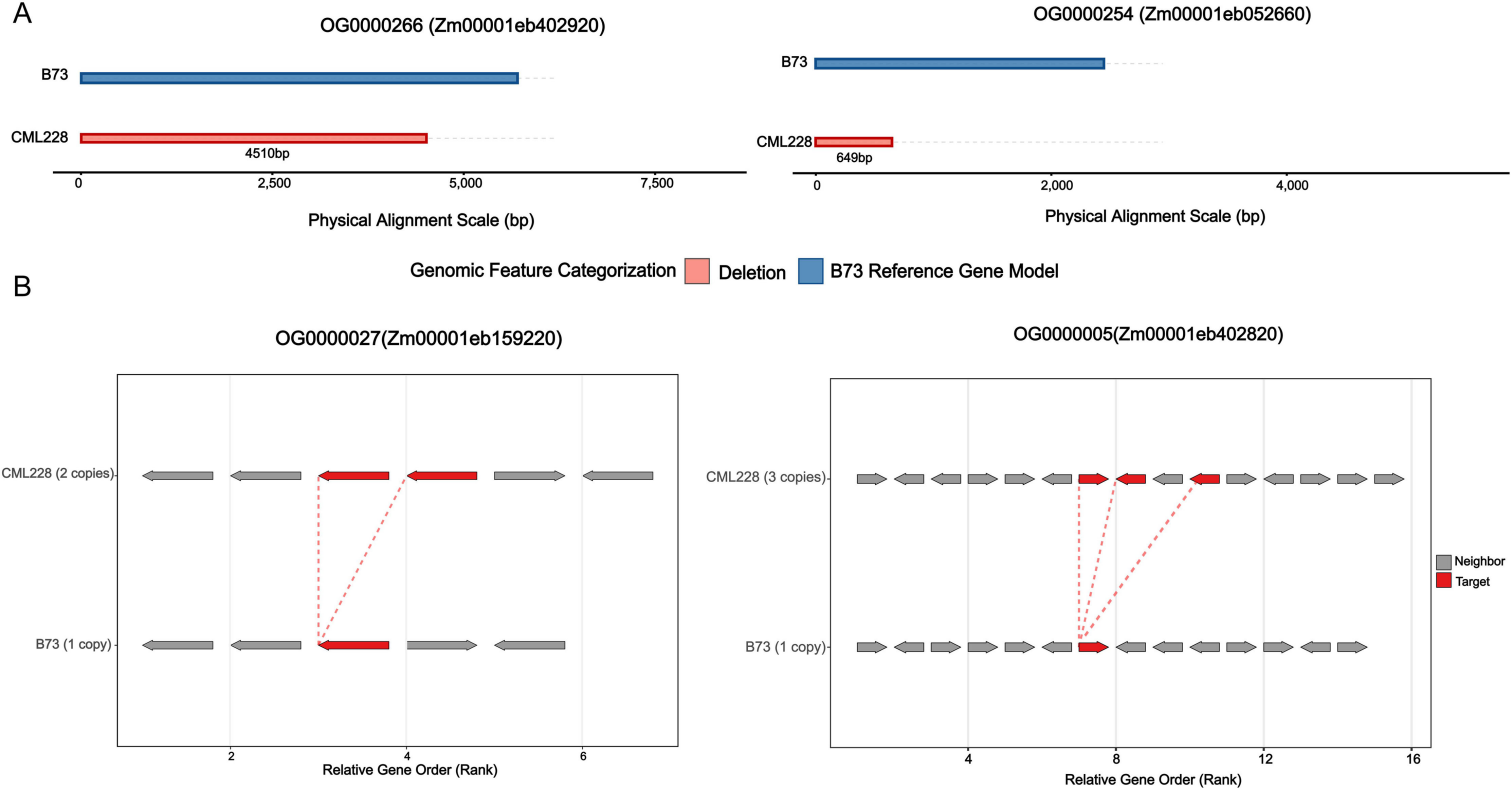
Structural validation of presence-absence variations (PAVs) and copy number variations (CNVs) for target orthogroups between B73 and CML228. **(A)** Physical alignment profiles of PAV-mediated gene disruptions in OG0000266 and OG0000254. Blue bars represent the B73 reference gene models, and red bars indicate the genomic deletion intervals identified in CML228, with respective deletion sizes (4,510 bp and 649 bp) mapped against the physical alignment scale (bp). **(B)** Micro-synteny and genomic neighborhood analysis validating gene copy number expansions in OG0000027 and OG0000005. The x-axis indicates the relative gene order (rank). Red and gray arrows represent the target genes and neighboring genes, respectively. Dashed lines trace orthologous relationships, illustrating the gene dosage transitions from a single copy in B73 to multiple copies (2 copies and 3 copies, respectively) in CML228.

To validate whether the observed PAV and CNV patterns reflect genuine genomic variation rather than annotation artifacts, we selected two representative PAV orthogroups and two representative CNV orthogroups for validation. Using SV data, we precisely mapped the coordinates of the target genes in representative varieties. The results showed that for the PAV loci, large-scale deletions covering the entire coding region were present in the accessions where the genes were absent. Specifically, OG0000266 (Zm00001eb402920) and OG0000254 (Zm00001eb052660) exhibited deletions of 4,510 bp and 649 bp on chromosomes 9 and 1, respectively, in CML228, leading to gene absence. For the CNV orthogroups (**Figure 3A**; **Supplementary Table 5**), OG0000005 (Zm00001eb402820) and OG0000027 (Zm00001eb159220) directly demonstrated genuine copy number variation: OG0000005 showed significant copy number expansion in CML228; while B73 carried only a single copy, CML228 harbored three tandemly arranged homologous copies within a conserved micro−synteny block, suggesting that this expansion originated from local tandem duplication. OG0000027 showed a modest copy number increase, with a single copy in B73 and two copies in CML228; both copies were located within a conserved micro−synteny block, and the neighboring homologous genes maintained a conserved arrangement, consistent with a recent local duplication event (**Figure 3B**, **Supplementary Table 6**). Collectively, these results indicate that the PAV and CNV patterns inferred from orthogroup occupancy are directly supported by genomic structural variation and reflect genuine genetic diversity within the maize pan−genome, rather than being caused by annotation differences. These validation analyses provide a reliable foundation for subsequent functional studies of structural variation and offer solid evidence for understanding the expansion and diversity of the *ZmCYP450* gene family.

### 3.2 Evolutionary mechanisms underlying the expansion and diversification of the *ZmCYP450* gene family

#### 3.2.1 Patterns of expansion and collinearity characteristics of the ZmCYP450 family driven by different types of duplication

**Figure 4:**
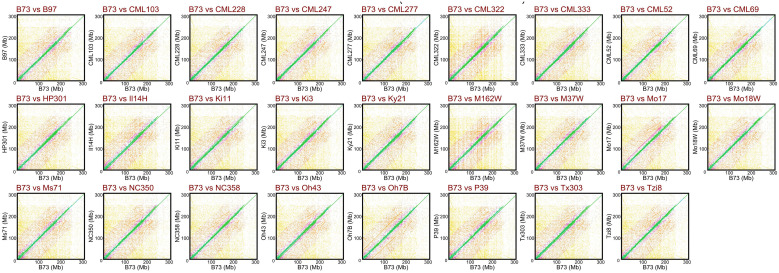
Collinearity and gene duplication patterns of the *ZmCYP450* genes in 27 Maize Varieties. Determination of *ZmCYP450* gene repeat type based on collinearity lattice. This figure illustrates the whole-genome synteny matrix between B73 and 26 maize varieties. WGD is marked in magenta, TRD in gold, TD in sky blue, PD in green, and DSD in orange; each point represents a duplicated gene, revealing the expansion pattern of the *ZmCYP450* gene family in maize.

**Figure 5:**
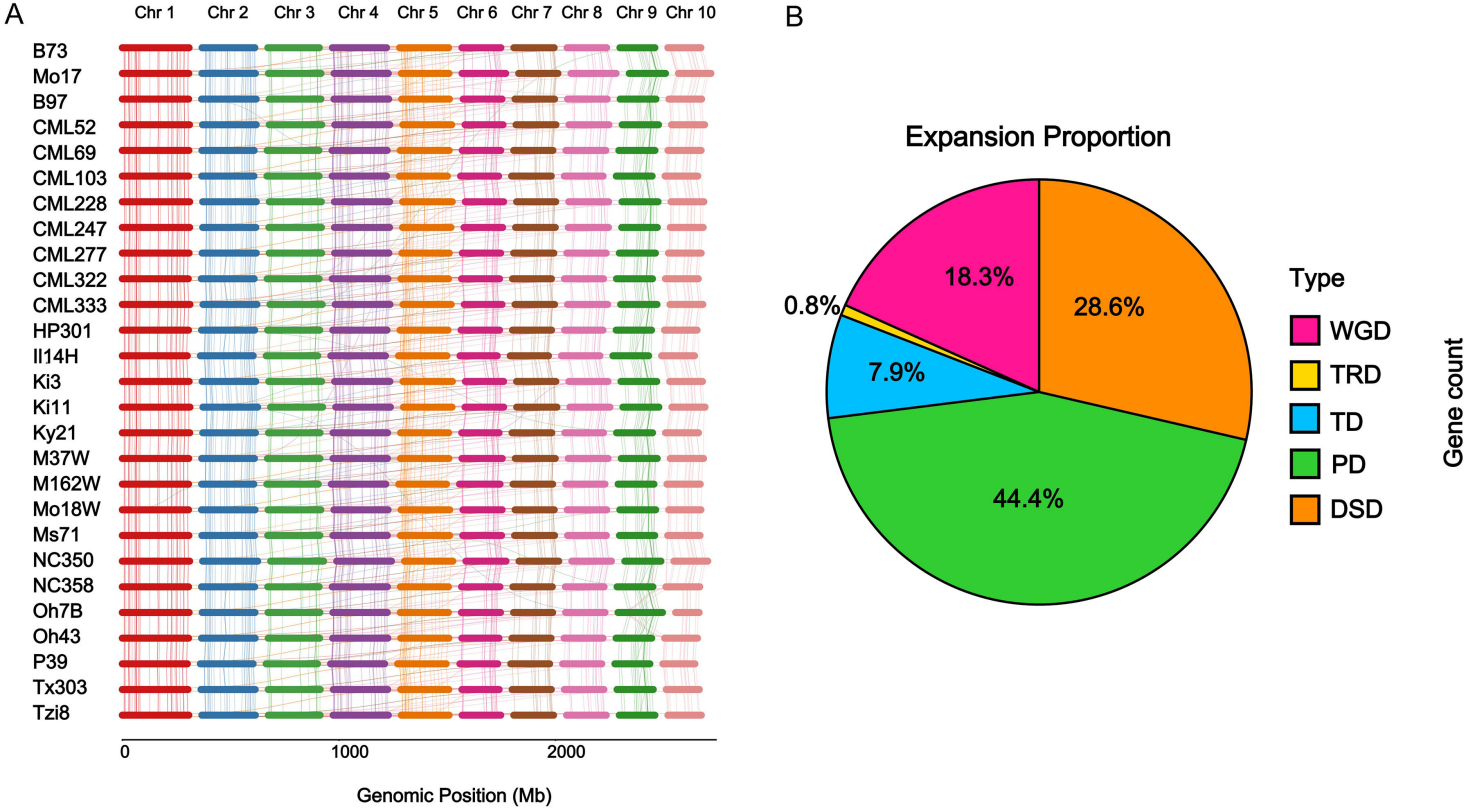
**(A)** Genomic Distribution and Homology of the *ZmCYP450* gene Family in 27 Maize Inbred Lines. Chromosomes are represented by horizontal, capsule-shaped bars, each of a different color, with their relative physical lengths plotted on the x-axis in Mb. These 27 maize genomes are arranged vertically, with the reference line B73 at the top. The colored lines (Bézier curves) represent homologous gene pairs identified between the reference line and the other varieties. **(B)** Pie chart of *ZmCYP450* gene duplication types by category.

Polyploidy events provide important genetic raw materials for biological evolution, promote plant genome rearrangement, and increase structural variation, thereby playing a key role in plant evolution (Wang et al., 2024). By analyzing the duplication origins of *ZmCYP450* genes, we classified them into different duplication types, including tandem duplication (TD), proximal duplication (PD), whole-genome duplication (WGD), dispersed duplication (DSD), and transposed duplication (TRD) (**Figure 4**). The results indicate that, across the entire genome, PD accounts for the highest proportion (44.4%), followed by DSD (28.6%), WGD (18.3%), and TD (7.9%), while TRD accounts for the lowest proportion (0.8%) (**Figure 5B**). Combining these findings with the genome-wide colinearity dot plot, we observed that TD and PD are primarily concentrated near the diagonal, exhibiting localized high-density clusters. In contrast, WGD and DSD display a more dispersed distribution pattern, spanning multiple chromosomal regions. Furthermore, TRD signals are primarily distributed far from the diagonal, exhibiting a punctate distribution across chromosomes or over long distances. Combining the various dot plots reveals distinct long colinearity segments, which may originate from historical whole-genome duplication events and have been preserved (Tang et al., 2008). Notably, in the B73 vs. Mo17 and B73 vs. Oh7B comparisons, we observed ultra-long WGD and DSD colinearity segments distinct from those in other combinations; these segments exhibit higher continuity and integrity.

Combined with collinearity analysis, it can be found that most *ZmCYP450* genes maintain a high degree of consistency in chromosome positions between different varieties, forming obvious horizontal collinearity blocks, especially on chromosomes such as chr1 and chr2. The collinearity of the lines shows high parallelism (**Figure 5A**). Although the overall collinearity framework remains stable, the gene order in certain regions exhibits subtle deviations. Oblique connections across chromosomes were observed on chromosome 2 of KI11, B97, and CML333, as well as on chromosome 9 of B73 and Mo18W. These patterns likely reflect minor local deviations in gene order between varieties, enriching intraspecific genomic variation in maize. In contrast, chr5 shows the most obvious oblique connection characteristics, and the collinearity relationship is the most complex.

#### 3.2.2 Divergent selection pressure characteristics among different orthogroups

**Figure 6:**
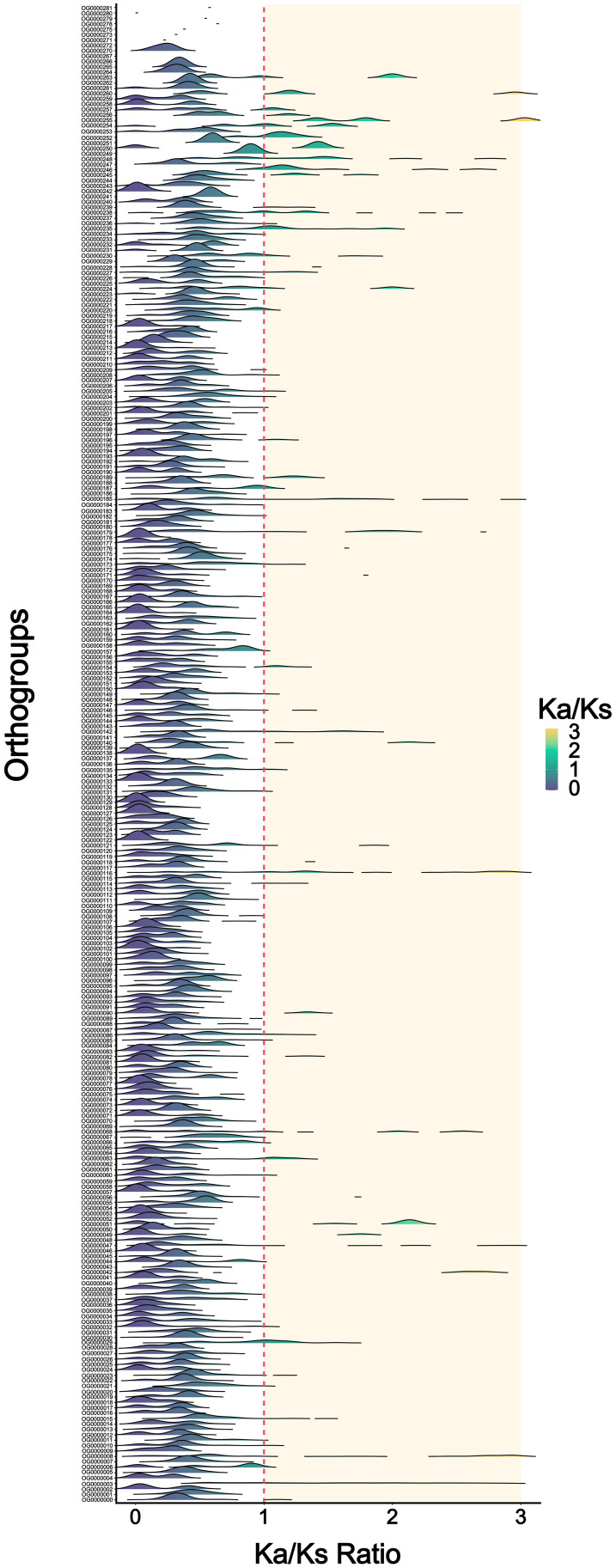
Kernel density plot. The kernel density plot illustrates the distribution of Ka/Ks ratios within each OGs. The x-axis represents the Ka/Ks ratio, while the y-axis lists the individual OGs. The red dashed line at Ka/Ks = 1.0 marks the threshold between purifying selection (Ka/Ks< 1.0) and positive selection (Ka/Ks > 1.0). The yellow shaded area highlights the OGs showing evidence of positive selection.

We calculated the Ka/Ks value of each ZmCYP450 OG based on the whole genome sequence of 27 maize varieties (**Figure 6**; **Supplementary Table 7**). In general, the Ka/Ks values of most OGs are significantly concentrated below 1, mainly distributed in the range of 0-0.5, and their density peaks are located in the lower value areas, indicating that the *ZmCYP450* gene family is strongly constrained by purifying selection during evolution, showing high functional conservation (Hurst, 2002). Nevertheless, the Ka/Ks values between different OGs still show significant differences. For example, the distribution curves of OGs represented by OG0000078, OG0000077, OG0000076 and OG0000075 are relatively narrow and concentrated, almost entirely located in the Ka/Ks< 1 range; in contrast, OGs represented by OG0000003, OG0000008, OG0000029 and OG0000068 showed a more dispersed distribution, and their Ka/Ks values could be extended to 1 or 2 or even more than 3, and formed a long tail or sub-peak on the right side of the neutral selection threshold (Ka/Ks = 1), suggesting that these genes underwent positive selection and showed more favorable mutations. Among the 282 OGs, only 73 OGs had a Ka/Ks value of more than 1, of which only 7 OGs exceeded 3, indicating that this phenomenon is not common in the ZmCYP450 family.

### 3.3 Phylogenetic relationships and subfamily diversification of the ZmCYP450 family

**Figure 7:**
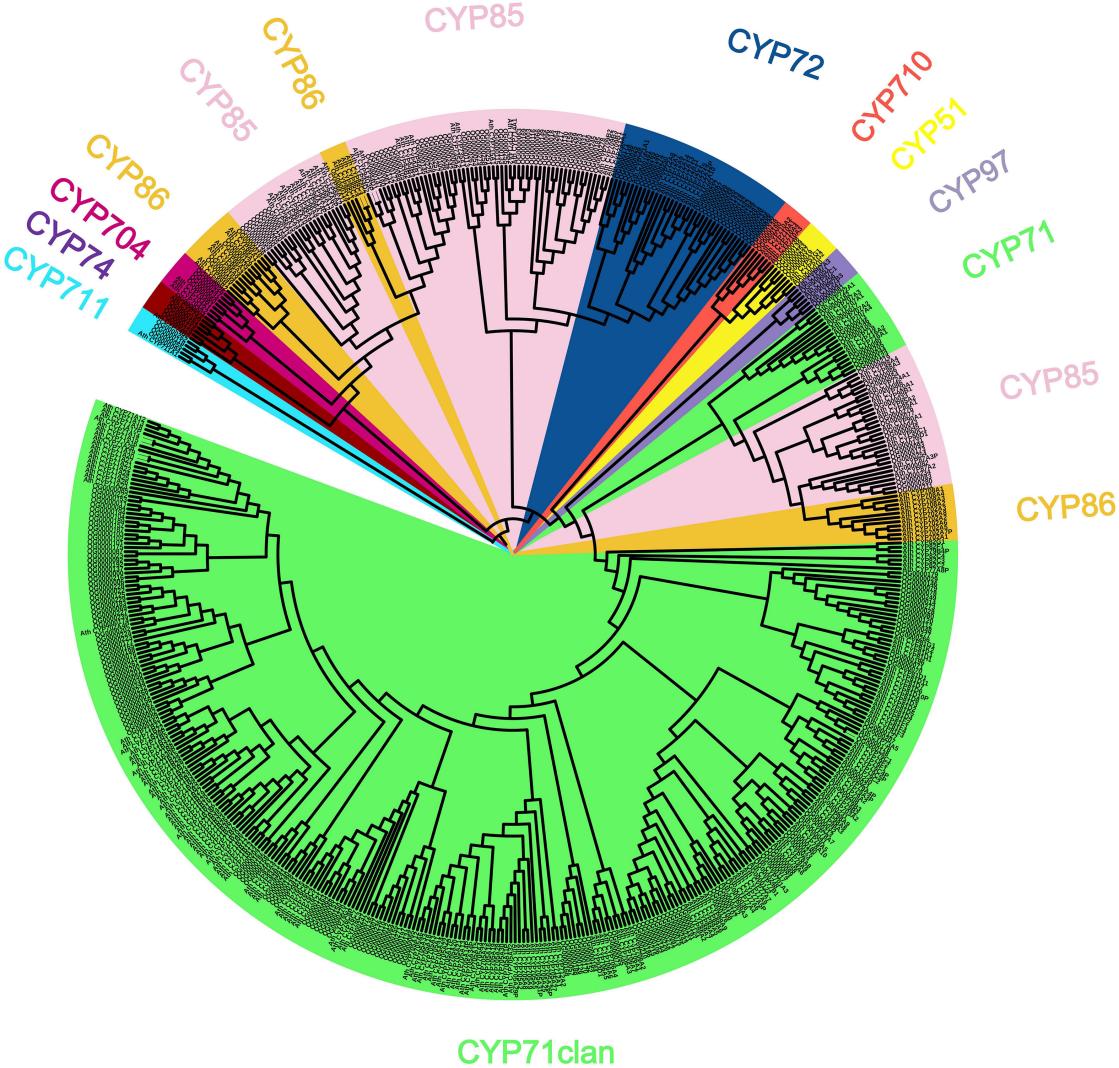
Phylogenetic tree of the representative CYP450 protein sequences from each orthogroup. The phylogenetic tree was constructed using IQ-TREE2 (v2.1.2) with 1,000 bootstrap repetitions. The phylogenetic tree was constructed using sequences from maize and *Arabidopsis*. The phylogenetic tree is divided into 10 clans, including CYP71, CYP86, CYP85, CYP97, CYP51, CYP710, CYP72, CYP704, CYP74, and CYP711.

To further investigate the classification and evolutionary relationships of the *ZmCYP450* gene family, we constructed a phylogenetic tree based on the 282 representative maize protein sequences, using 272 *Arabidopsis thaliana* CYP450 sequences as a reference (**Figure 7**, **Supplementary Table 8**). In line with previous research, we clearly classified the *CYP450* genes into 10 clans, with the CYP71 clan being the largest branch, comprising 319 genes and representing the most abundant group in maize. This branch primarily includes multiple families such as CYP71, CYP81, CYP82, CYP83, CYP75, and CYP89, constituting the main portion of the phylogenetic tree. Apart from the CYP71 clan, the remaining *ZmCYP450* genes are primarily distributed across several conserved clans. Among these, the CYP85 clan includes families such as CYP85, CYP90, CYP88, and CYP724, constituting the second-largest group; the CYP72 clan encompasses families such as CYP72, CYP734, CYP735, CYP714, and CYP709, forming another significant branch. The remaining maize *CYP450* genes are distributed across several smaller clans, including CYP710, CYP86, CYP704, CYP74, CYP711, CYP97, and CYP51. The number of maize genes in these branches is relatively small, and they typically form one-to-one or small-scale clustering relationships with their *Arabidopsis* homologs. Among them, the CYP711 clan is the smallest, containing only five genes. Notably, unlike the concentrated expansion patterns observed in other clans, the CYP86 and CYP85 clans exhibit a more dispersed expansion pattern.

A cross-referential analysis integrating selection pressure profiles (**Figure 6**) with the phylogenetic framework (**Figure 7**) revealed that the distribution of these 73 orthogroups undergoing positive selection across different clans was not random, but rather exhibited a pronounced tendency. These OGs, which exhibit pronounced expression and sequence divergence (suggestive of potential functional divergence), were predominantly concentrated within the most massively expanded CYP71 clan, as well as the CYP85 and CYP72 clans that are closely associated with phytohormone metabolism. Given that the CYP71 clan is extensively involved in the biosynthesis of specialized (secondary) metabolites in maize, while the CYP85 and CYP72 clans primarily govern phytohormone homeostasis and developmental regulation, this evolutionary preference toward specific functional categories may have important biological implications. Notably, a substantial proportion of the OGs undergoing positive selection were represented by sequences from the B73 and Mo17 genomes in the phylogenetic tree. On one hand, this phenomenon can be attributed to the exceptionally high annotation quality and sequence integrity of genomes such as B73 and Mo17, which renders them ideal reference templates for accurately calculating evolutionary pressures (Sun et al., 2018). On the other hand, it profoundly reflects that the rapid evolution of these specific ZmCYP450 OGs is likely a direct consequence of intensive artificial selection pressure during modern agricultural breeding, aimed at optimizing elite agronomic traits such as high yield and multi-resistance (Fontes-Puebla et al., 2021).

### 3.4 Mechanisms of functional diversification in the *ZmCYP450* gene driven by structural variation

#### 3.4.1 Structural variation-mediated gene loss and gene integrity alteration

**Figure 8:**
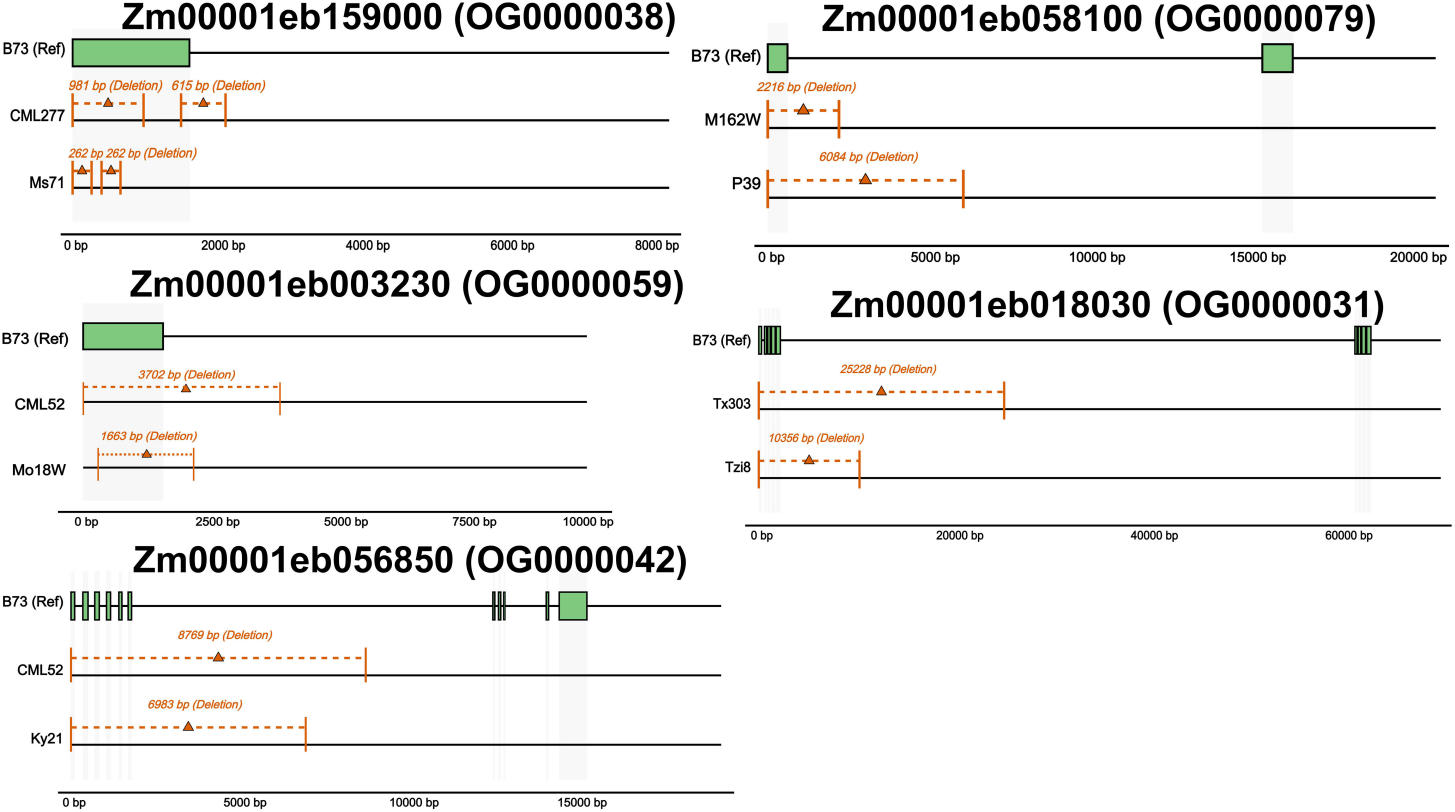
Representative gene structure variations in the ZmCYP450 family. This figure illustrates the structural variations in the selected genes within the maize genome, which manifest as large-scale deletions. Compared to the B73 reference line, multiple varieties exhibit significant deletions in the coding region of the *ZmCYP450* gene or within a 2-kb region upstream or downstream of it (indicated by orange dashed lines).

To investigate the potential impact of structural variations (SVs) on the function of *ZmCYP450* genes, we employed a hierarchical screening strategy. First, based on the SV profiles of 27 maize accessions, we identified candidate genes harboring SVs within their coding or promoter regions. To prioritize representative targets for in-depth functional analysis from the gene family, we utilized MUMmer (v4.0.0beta2) to identify SVs exceeding 50 bp that overlapped with critical exons or core promoter regions. Following this, five *ZmCYP450* genes (Zm00001eb018030, Zm00001eb159000, Zm00001eb056850, Zm00001eb003230, and Zm00001eb058100) were identified as being significantly impacted by SVs. To visualize the distinct variation patterns, we performed structural comparisons for each gene by selecting the accessions exhibiting the maximum and minimum degree of SVs (Max-SV and Min-SV varieties, respectively) (**Figure 8**; **Supplementary Table 8**). The results showed that, compared to the reference genome B73, the primary structural variation types at these five loci were deletions of varying sizes. Among them, Zm00001eb018030 (belonging to the soft-core group OG0000031) exhibited the largest deletion event among the analyzed loci: a large-scale deletion of approximately 25,228 bp was present in Tx303, with the entire promoter and core coding region lost; a 10,356 bp deletion was also detected in Tzi8, indicating significant structural differences at this locus across different varieties. In the flanking or potential regulatory regions, Zm00001eb056850 (belonging to the core group OG0000042) and Zm00001eb058100 (belonging to the core group OG0000079) also exhibited large-scale deletions; for example, the former was deleted by 8,769 bp and 6,983 bp in CML52 and Ky21, respectively, while the latter exhibited a significantly larger deletion in P39 (6,084 bp) compared to M162W (2,216 bp), suggesting that large deletion events within regulatory regions may influence gene transcription. Furthermore, Zm00001eb003230 (belonging to the core group OG0000059) and Zm00001eb159000 (belonging to the core group OG0000038) further reveal the direct impact of SVs on coding regions. In Zm00001eb003230, the 3,702 bp deletion occurring in CML52 completely covered the promoter and the entire coding region, indicating more severe structural disruption compared to Mo18W (1,663 bp). In Zm00001eb159000, the deletions in the coding region exhibited polymorphism; for example, CML277 contained multiple deletion fragments, whereas Ms71 had only a small-scale deletion and maintained a high degree of conservation. These results indicate that SVs can directly disrupt gene structure, thereby affecting gene function.

#### 3.4.2 Protein truncation and loss of functional domains caused by structural variants

**Figure 9:**
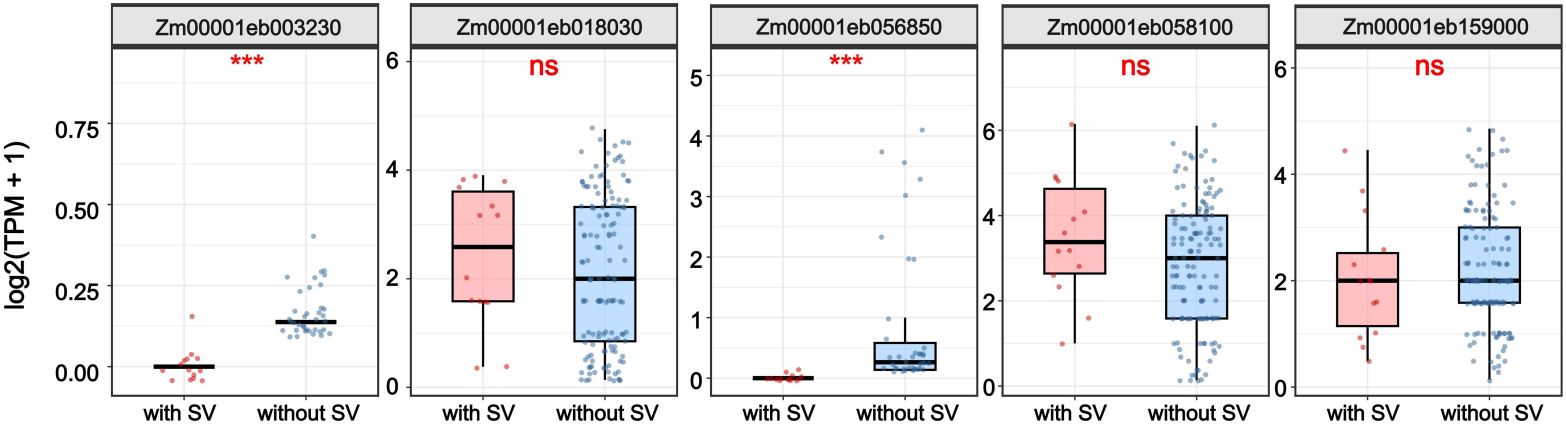
Impact of structural variations (SVs) on the expression levels of five candidate *ZmCYP450* genes. Expression profiles across five core tissues (leaf, shoot, root, tassel, and ear) are displayed. To ensure high data quality and transcriptomic statistical reliability, varieties exhibiting zero or undetectable transcript expression levels for a specific gene across the monitored tissues were rigorously filtered out prior to statistical evaluation. For a standardized comparative framework, the representative baseline sample size across the candidate loci is designated as *n* = 2 vs 20 independent maize varieties (with SV vs. without SV, respectively), where the statistical unit is defined as an individual maize line. To fully account for all data points plotted in the jittered distribution, the sample size is further specified as *n* = 10 vs 100 merged tissue observations (representing 2 lines × 5 tissues for the SV-positive group, versus 20 lines × 5 tissues for the control group). Significant differences between groups were evaluated by the non-parametric Wilcoxon rank-sum test, and the resulting *P*-values were adjusted for multiple-testing using the Benjamini-Hochberg (BH) method. Exact adjusted *P*-values for each facet are: *Zm00001eb003230* (*P*_adj_*=* 5.54×10^-5^), Zm00001eb018030(*P*_adj_*=*0.752), *Zm00001eb056850* (*P*_adj_*=* 3.09×10^-7^), Zm00001eb058100(*P*_adj_*=*0.752), *and Zm00001eb159000* (*P*_adj_*=*0.901). Statistical significance is indicated by *** (*P*_adj_< 0.001), and ns means not significant.

**Figure 10:**
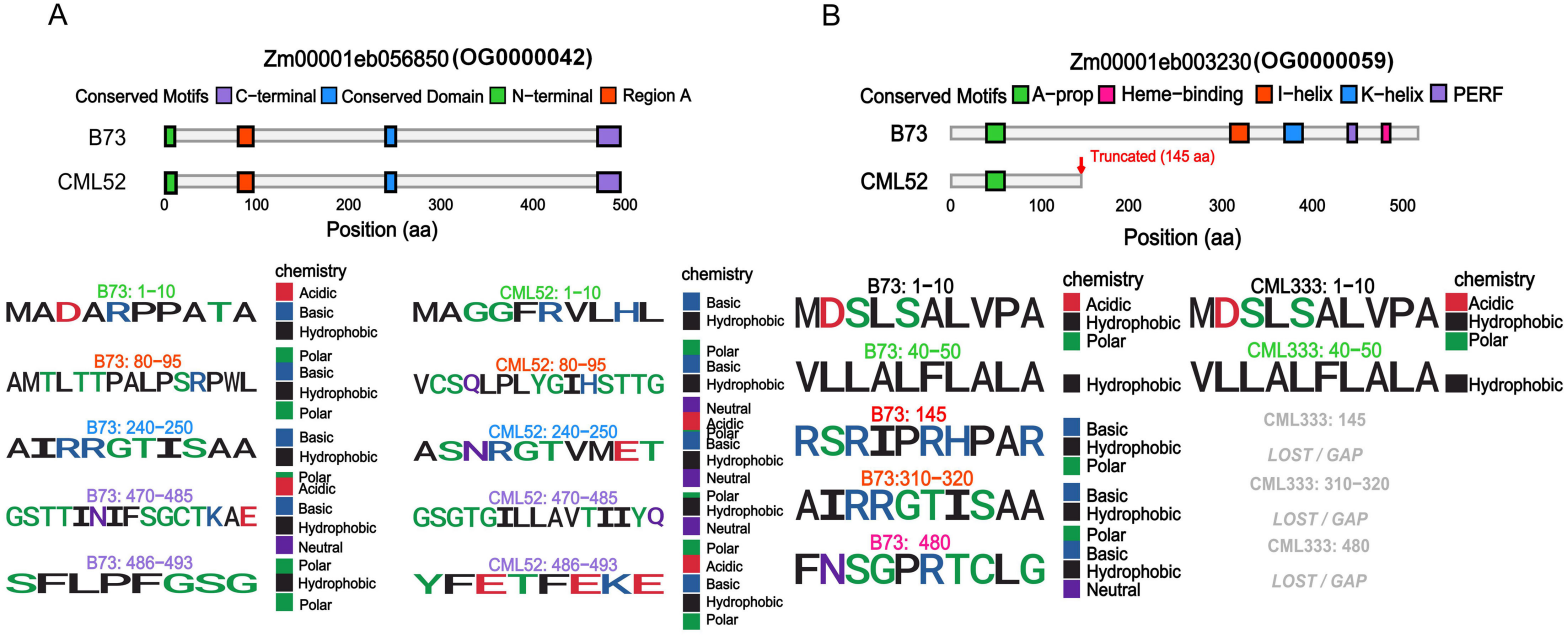
The effects of structural variations (SVs) on protein structure. **(A)** The upper part of the figure shows the distribution of conserved motifs in Zm00001eb056850 (OG0000042); the lower part, using WebLogos, highlights significant changes in the physicochemical properties of amino acids at specific sites. **(B)** The upper part of the figure shows five key conserved motifs of Zm00001eb003230 (OG0000059). The red arrow indicates that the gene undergoes premature termination at approximately 145 amino acids and lacks four of the five conserved motifs. The lower part of the figure displays a WebLogo alignment of this gene across different strains. All functional motifs downstream of position 145 are completely absent in CML52 (marked as ‘*LOST/GAP*’).

After defining the structural variation (SV) patterns of these five representative genes, we further integrated multi-tissue transcriptomic data to evaluate whether these DNA-level rearrangements result in actual changes in transcriptional levels. We analyzed the expression divergence between accessions with and without SVs overlapping these genes. The results revealed that, among the five candidate genes, only Zm00001eb003230 and Zm00001eb056850 exhibited highly significant expression differences (*P*_adj_ < 0.001) (**Figure 9**). Specifically, these two genes showed significantly reduced or nearly undetectable expression in accessions harboring SVs, while maintaining relatively high transcript levels in those without, suggesting that these large-scale SVs exert a strong inhibitory effect on their transcriptional activity. Given that functional gene alteration requires substantial changes in transcription and translation, our subsequent in-depth mechanistic analysis—focusing on the integrity of protein core domains and the reorganization of cis-regulatory elements—will strictly concentrate on these two genes with confirmed expression changes. After confirming that structural variants (SVs) significantly suppress the transcriptional expression of Zm00001eb003230 and Zm00001eb056850, we further evaluated their potential impact on gene function at the protein structural level. Through comparison of protein sequences and domain annotation analysis between the reference variety B73 and representative mutant varieties, the results showed that in Zm00001eb056850, the protein length between B73 and CML52 remained generally consistent (**Figure 10A**), and typical CYP450 conserved domains (such as the I-helix, K-helix, and heme-binding region) were all fully preserved, indicating that this gene has not undergone structural disruption during evolution. However, local sequence alignment (WebLogo) revealed multiple sites of non-synonymous substitutions in CML52 compared to B73 within the 240–250 aa and 470–485 aa regions. Although these changes did not disrupt the overall protein structure, they may affect protein stability; such minor mutations can alter biochemical function and drive further evolution (Bloom et al., 2006). In contrast, Zm00001eb003230 exhibits a completely different pattern of variation (**Figure 10B**). In CML52, the gene exhibits a premature termination at approximately 145 aa, resulting in a significantly shortened protein length that retains only the N-terminal region. Furthermore, the truncated protein retains only the A-prop region, while critical catalytic domains such as the I-helix, K-helix, PERF motif, and heme-binding regions are completely absent. The corresponding sequence alignment results also show that all functional regions downstream of the truncation site are completely lost, represented in the figure as a continuous ‘*LOST/GAP*’, which predicts that this type of truncation likely leads to complete protein inactivation.

#### 3.4.3 Restructuring of cis-regulatory elements driven by promoter structural variations

**Figure 11:**
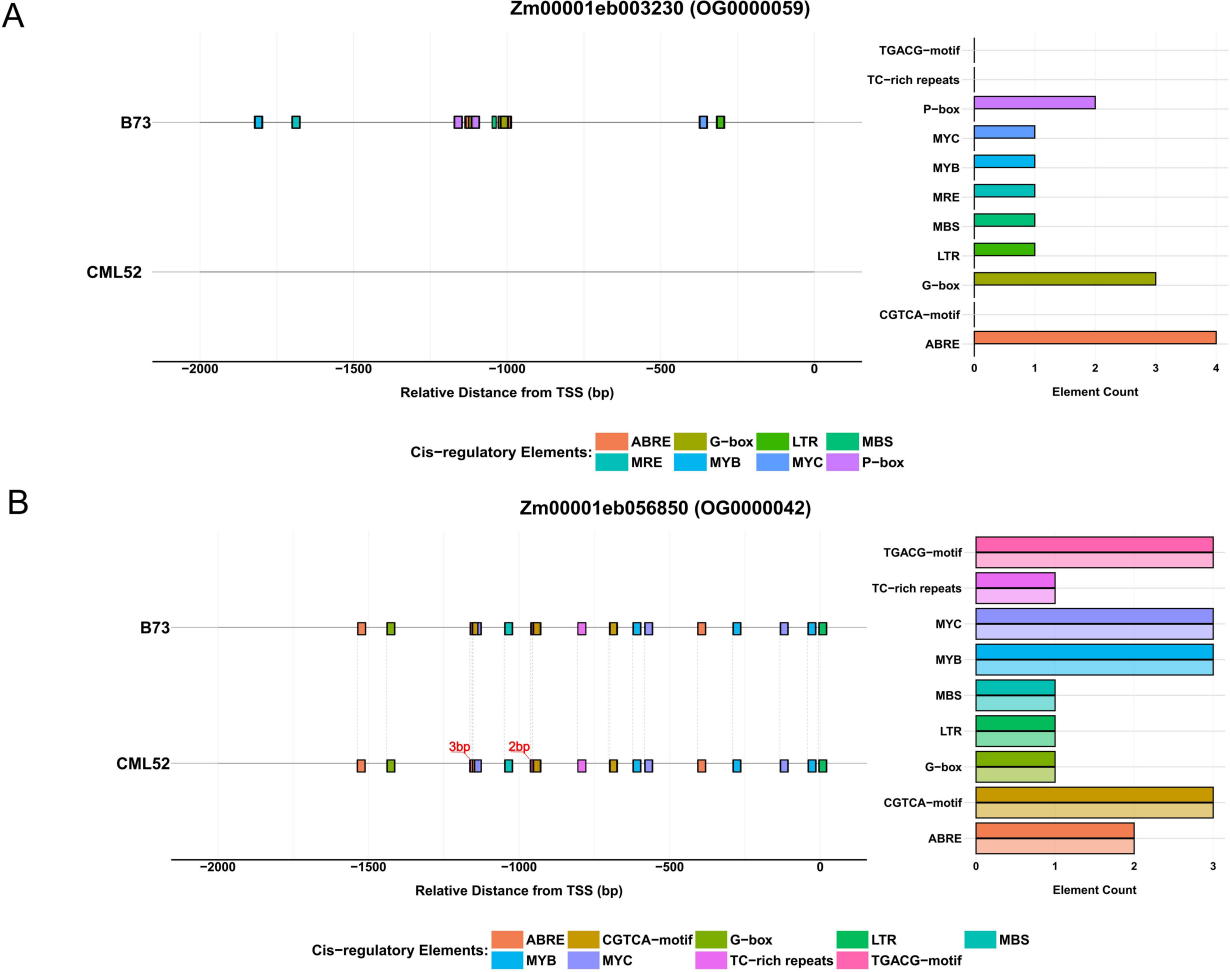
The Effects of Structural Variations (SVs) on Promoter Regions. The figure on the left shows the spatial distribution of cis-regulatory elements within the 2,000 bp region upstream of the transcription start site (TSS); the bar chart is color-coded according to the functional categories defined in the legend at the bottom. **(A)** In Zm00001eb003230, regulatory elements are completely absent in CML52 compared to the B73 reference. **(B)** In Zm00001eb056850, the promoter structure between B73 and CML52 is relatively conserved and contains various stress-related and hormone-responsive elements (such as ABRE, the CGTCA motif, and MYC).

Further promoter analysis revealed that the promoters of both genotypes contain various classical regulatory elements, including light-responsive elements (G-box), hormone-responsive elements (ABRE), stress-responsive elements (MBS, LTR), and transcription factor binding sites (MYB, MYC). In Zm00001eb003230, the SVs caused significant disruption to the structure of the CML52 promoter, particularly the loss of core promoter elements (**Figure 11A**). In contrast, the promoter region of Zm00001eb056850 did not exhibit large-scale sequence deletions; instead, the distribution of cis-acting elements was fine-tuned through changes in the spacing between elements (**Figure 11B**). Compared to B73, CML52 showed no significant changes in the number of hormone-responsive (ABRE, ERE) and stress-responsive (LTR, MBS) elements, nor in the number of light-responsive (G-box) elements. Overall, structural variations regulate gene transcriptional activity through either the direct loss of core cis-regulatory elements or the rewiring of their spatial distribution (Qi et al., 2025).

### 3.5 Genomic distribution and transcriptional activity of typical and atypical genes

**Figure 12:**
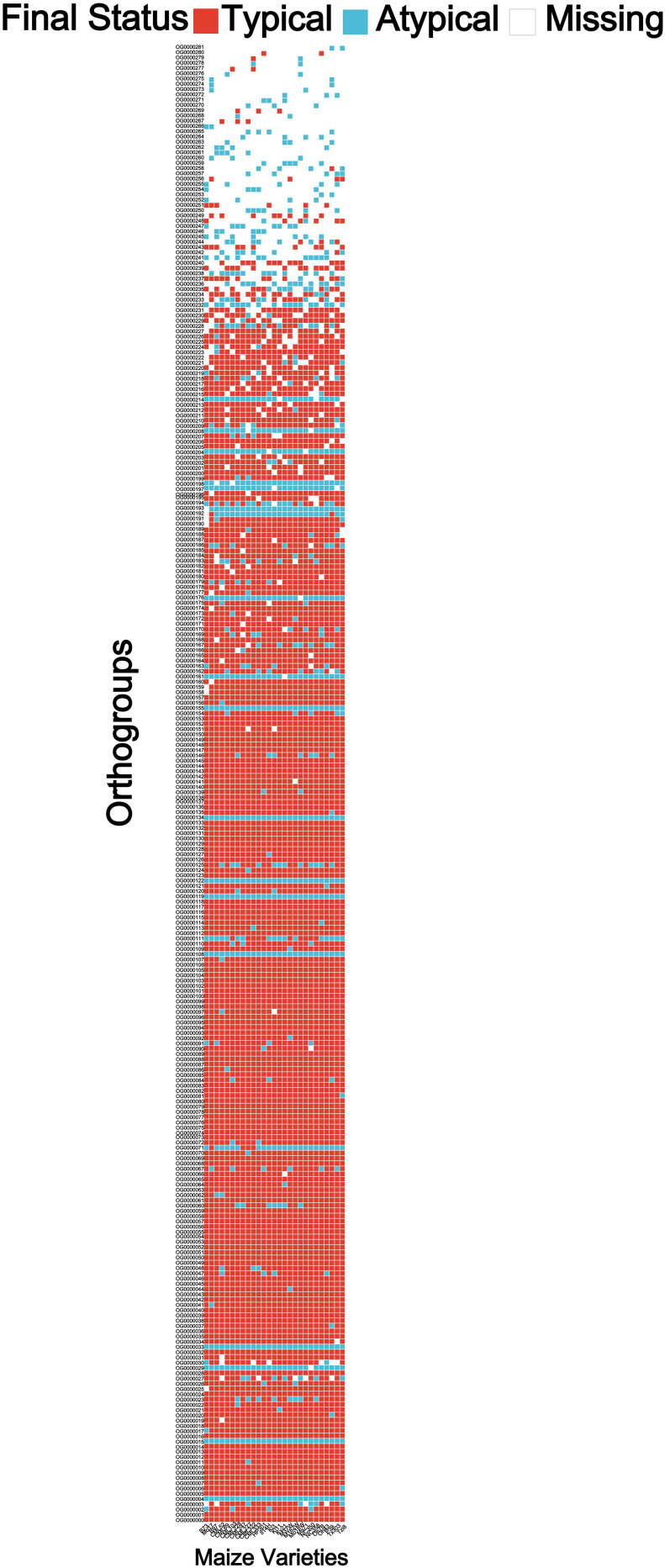
Widespread expression of typical and atypical *ZmCYP450* genes in maize. The heatmap shows the classification of each *ZmCYP450* gene across different maize varieties as either a ‘Typical’gene an ‘Atypical’ gene, or ‘Missing’.

**Figure 13:**
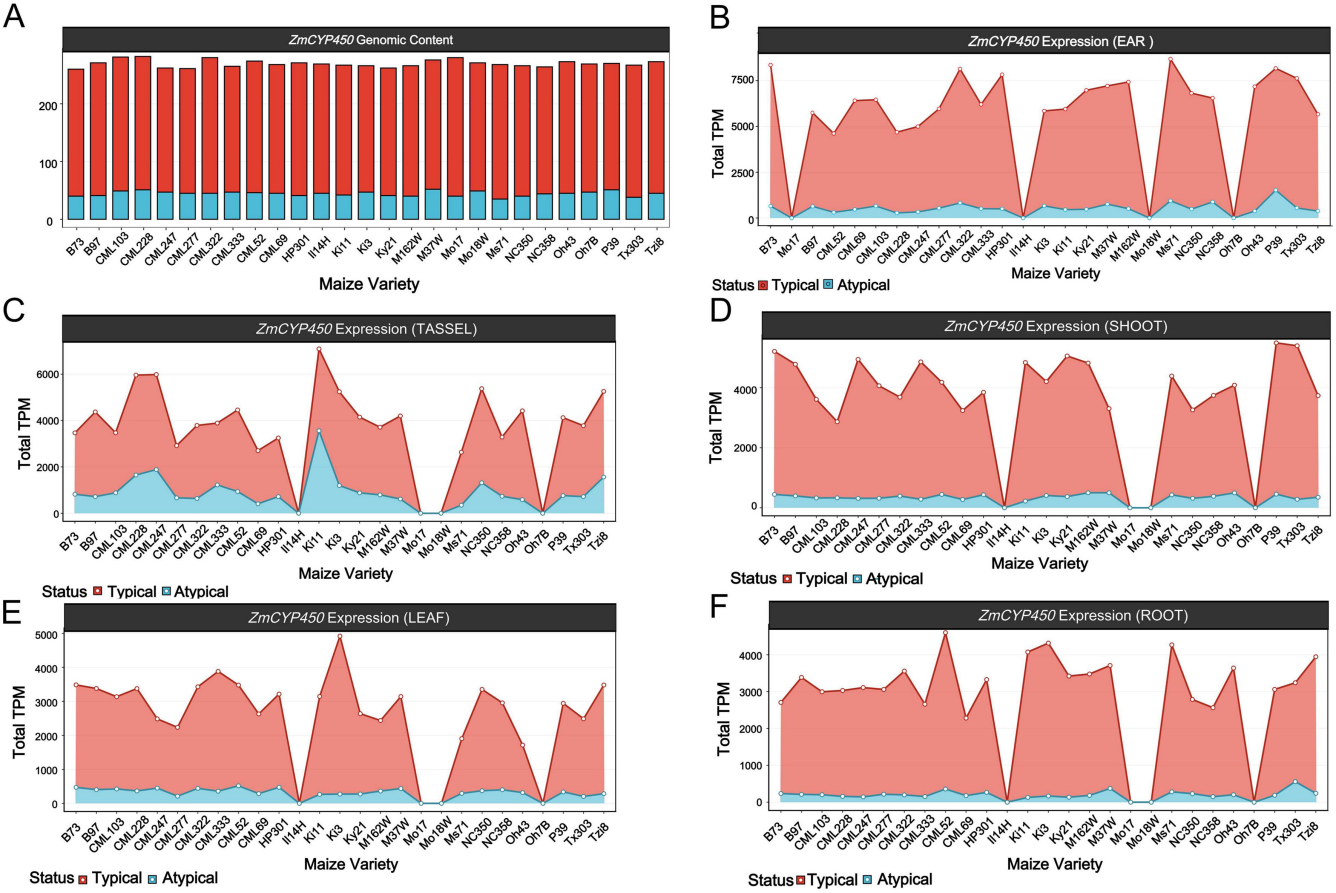
**(A)** The image shows the number of typical and atypical genes in each maize variety. **(B–F)** The image displays the expression levels of typical and atypical genes across five tissues (leaf, shoot, root, tassel, and ear) in different maize inbred lines.

To investigate the impact of structural integrity on the function of *ZmCYP450* genes, we classified ZmCYP450 members into structurally intact typical genes (Typical, red), mutated and truncated atypical genes (Atypical, blue), and complete gene loss events (Missing, white) (**Figure 12**). The results indicate that the vast majority of *ZmCYP450* genes belong to the structurally intact typical members, and all maize varieties retain the P450 core catalytic domain. Within numerous orthogroups, we observed a distinct distribution pattern characterized by alternating mosaics of typical, atypical, and missing genes. The high frequency of atypical states and complete loss events may be attributed to sequence truncation or locus loss caused by large-scale structural variations (SVs). To validate the impact of typical and atypical genes on the biological functions of the ZmCYP450 family, this study further integrated genomic structural data with transcriptomic data from five representative tissues (leaf, shoot, root, tassel, and ear) to conduct a systematic analysis of the expression patterns of the ZmCYP450 family across 27 maize varieties (**Figure 13**). At the genomic level, the number of ZmCYP450 family members showed relative conservation across the 27 varieties, with each variety retaining more than 250 gene members. Among these, structurally intact typical genes constitute the backbone of the family’s core function, while atypical genes affected by structural variations maintained a stable proportion of approximately 40 to 60 copies across varieties. In stark contrast to this genomic stability, the overall expression levels of the ZmCYP450 family exhibited dramatic dynamic fluctuations across varieties and tissues. For instance, varieties such as B73, Ki3, and Ki11 displayed extremely high transcriptional activity, whereas varieties including II14H, Mo17, and Oh7B exhibited extremely low expression levels in multiple tissues, approaching zero. Although the genomic copy numbers of atypical genes were significantly lower than those of typical genes in all examined tissues, they contributed a non-negligible proportion to the total TPM values. It is worth noting that in tassel tissues, the proportion of atypical gene expression in varieties such as Ki11, Ki3, and CML247 was significantly elevated. This result strongly demonstrates that sequence truncation caused by structural variations does not lead to complete transcriptional silencing of *ZmCYP450* genes.

**Figure 14:**
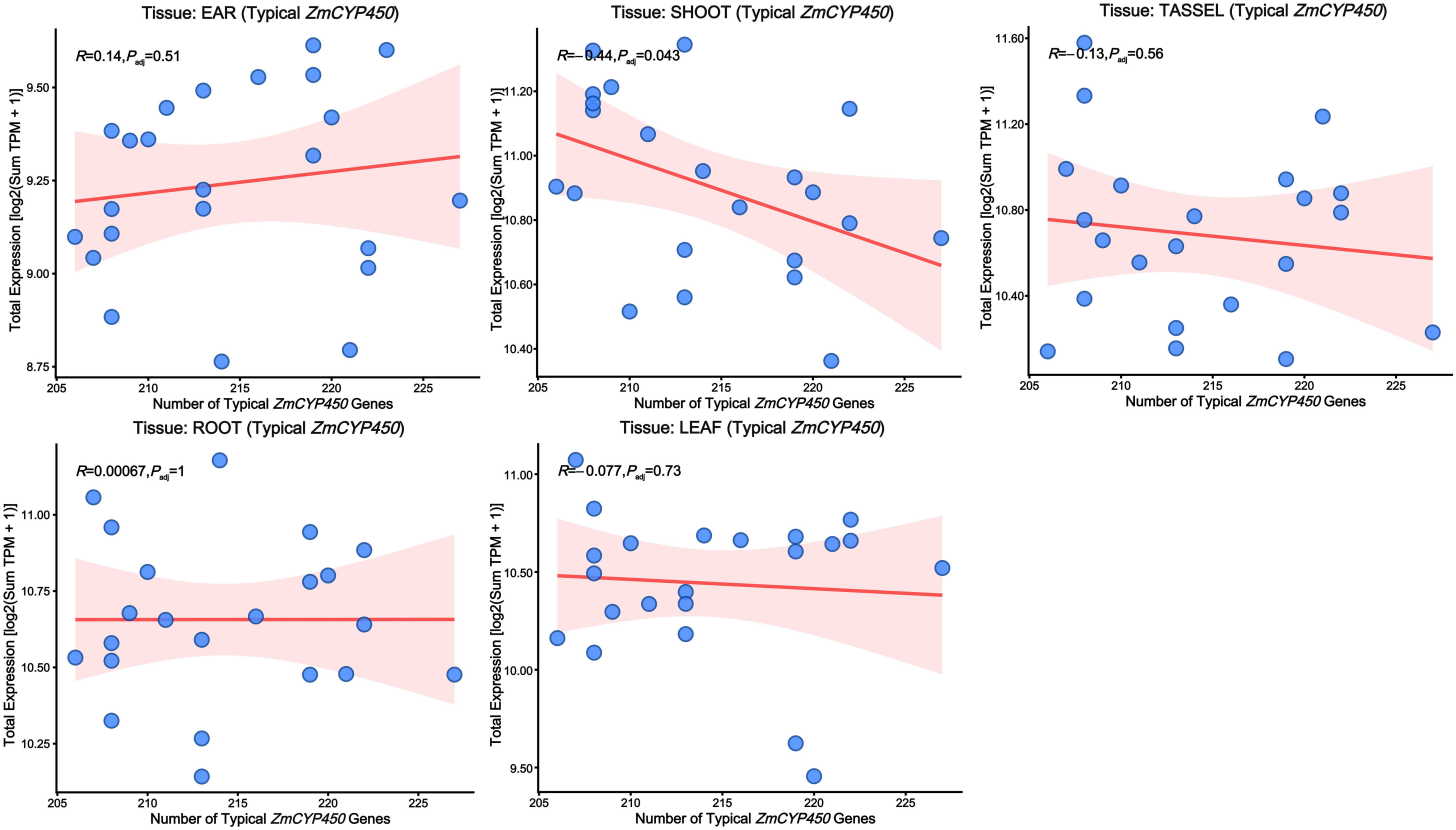
Analysis of the correlation between *ZmCYP450* gene count and total expression level per tissue. Linear regression analyses between the number of structurally complete *ZmCYP450* genes and the overall family expression level across five major maize tissues. To ensure data quality and avoid potential technical artifacts, maize varieties with zero or undetectable total transcript abundance in a given tissue were excluded prior to statistical analysis. The resulting analyses are shown separately for ear (n = 23 lines), tassel (n = 22 lines), leaf (n = 22 lines), root (n = 22 lines), and shoot (n = 22 lines). The x-axis represents the number of structurally complete *ZmCYP450* genes in each genome, and the y-axis represents the log_2_-transformed total expression level of the ZmCYP450 family [log_2_(Sum TPM + 1)] within the corresponding tissue. The red solid line indicates the fitted linear regression, and the shaded area denotes the 95% confidence interval. Pearson correlation coefficients (*R*) and Benjamini–Hochberg adjusted *P*-values (*P*_adj_) are shown in each panel. Raw *P*-values were corrected for multiple testing using the Benjamini–Hochberg false discovery rate (*FDR*) procedure. Statistical significance was defined as *P*_adj_< 0.05.

To further investigate the relationship between gene structural integrity and expression level, this study examined the correlation between the number of typical genes in different maize varieties and the overall expression level of the gene family (**Figure 14**). In the above−ground tissues shoot (*R* = –0.44, *P*_adj_ = 0.043), leaf (*R* = –0.077, *P*_adj_ = 0.73), and tassel (*R* = –0.13, *P*_adj_ = 0.56), the number of typical genes showed a weak negative correlation with total expression level, with mean TPM values all exceeding 8.5. In contrast, ear (*R* = 0.14, *P*_adj_ = 0.51) and root (*R* = 0.00067, *P*_adj_ = 1) exhibited a weak positive trend, suggesting that an increased number of typical genes in these tissues may, to some extent, elevate tissue−specific expression levels. Notably, a significant negative correlation was detected only in the shoot tissue (*R* = –0.44, *P*_adj_ = 0.043), indicating that an increase in the number of typical genes in this tissue is significantly associated with a decrease in total expression level.

### 3.6 Expression landscapes and tissue-specific patterns of *ZmCYP450* genes

#### 3.6.1 Expression landscapes of *ZmCYP450* genes across tissues and maize varieties

**Figure 15:**
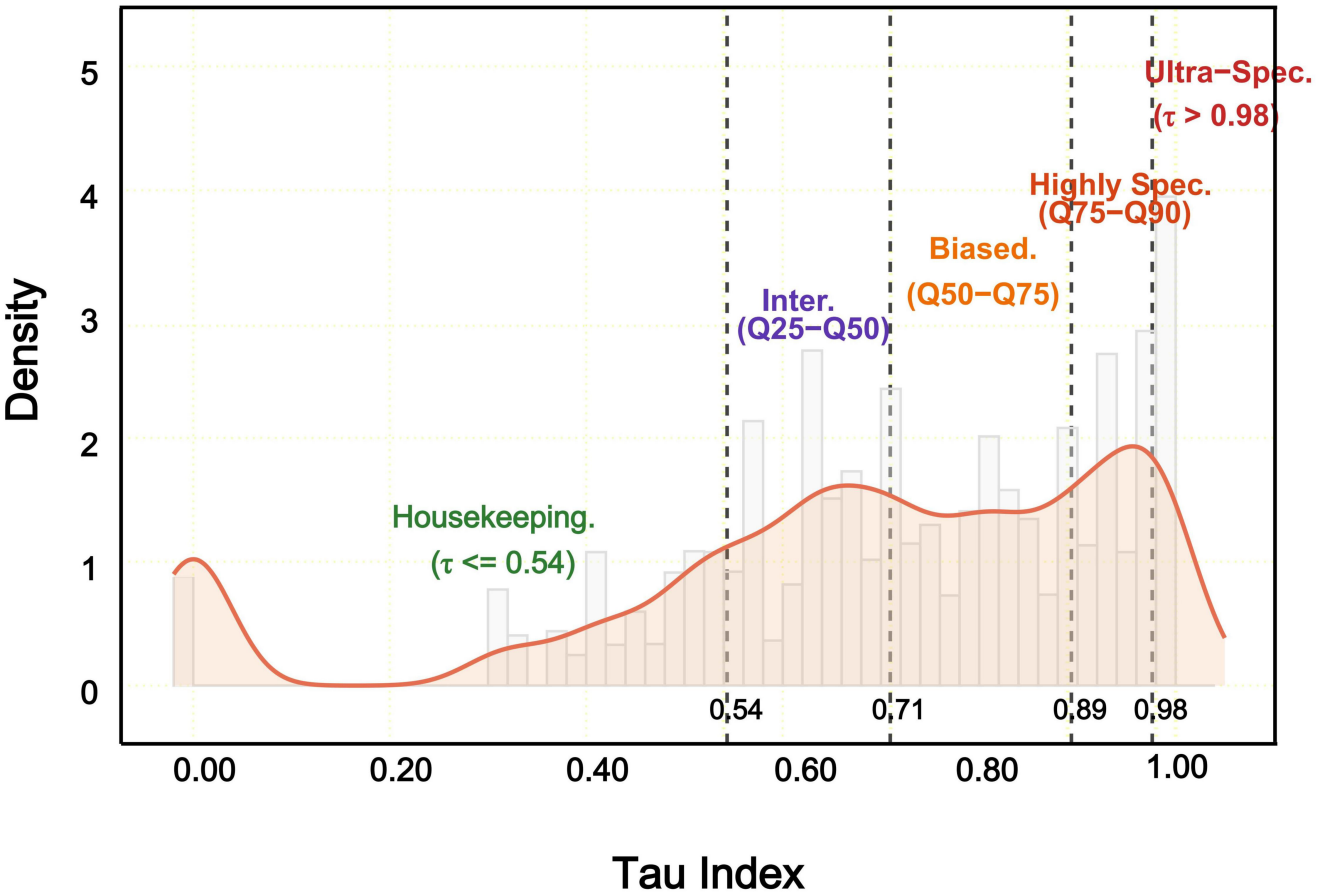
Classification of *ZmCYP450* genes based on tissue specificity index (τ). This figure shows the distribution of τ values for members of the analyzed gene family. A red dashed line indicates the threshold used to classify genes into the housekeeping, intermediate, tissue-biased, highly specific, and ultra-specific groups. The x-axis represents the tissue specificity index (τ), and the y-axis represents the density of genes within each interval; the distribution peaks are shifted to the right.

To characterize expression specificity, all *ZmCYP450* genes were classified using the tissue specificity index (τ) and a data-driven percentile-based threshold strategy (**Figure 15**). The distribution of τ values was markedly right-skewed, with a median value of 0.71 (**Supplementary Table 9**), indicating that tissue-specific expression is a prevalent feature of the ZmCYP450 family. Based on the quartile distribution of τ values, genes were classified into five categories: Housekeeping (τ ≤ 0.54), Intermediate (0.54< τ ≤ 0.71), Tissue-biased (0.71< τ ≤ 0.89), Highly specific (0.89< τ ≤ 0.98), and Ultra-specific (τ > 0.98).

Subsequently, a systematic expression stratification analysis was conducted for 282 ZmCYP450 orthogroups (OGs) based on their transcript abundance (TPM) across five major tissues and the tissue specificity index (τ) (**Supplementary Figure 1A**). Overall, distinct expression gradients were observed among different τ categories. Housekeeping OGs (τ ≤ 0.54) generally exhibited broad expression patterns, with transcripts distributed across leaf, shoot, root, tassel, and ear tissues without a clearly dominant expression site. For example, OG0000008 maintained relatively high expression levels in all five tissues, representing a typical broadly expressed pattern. As τ values increased, Intermediate OGs gradually displayed greater expression divergence among tissues, although they still retained multi-tissue expression characteristics. In the Tissue-biased category (0.71< τ ≤ 0.89), tissue preference became more pronounced. Some OGs were predominantly expressed in roots, such as OG0000131 and OG0000204, whereas others showed markedly elevated expression in ears. For instance, OG0000225 exhibited substantially higher expression in ear than in the other tissues. Meanwhile, several OGs remained preferentially expressed in leaves, indicating a clear trend of expression divergence within the ZmCYP450 family. With further increases in τ values, Highly specific and Ultra-specific OGs exhibited increasingly restricted expression patterns, with transcript accumulation concentrated in a limited number of tissues or even a single tissue, reflecting strong tissue specificity. These OGs generally maintained very low expression levels in most tissues but showed pronounced expression peaks in specific tissues. For example, OG0000101, OG0000103, and OG0000104 were predominantly expressed in shoot, whereas OG0000060 was almost exclusively enriched in tassel and exhibited minimal expression in the remaining tissues. Overall, the expression landscape of the ZmCYP450 family displayed a gradual transition from broad expression to highly tissue-specific expression, reflecting progressive expression specialization and potential functional differentiation across different maize tissues.

To further investigate the expression stability and variation patterns of genes across different functional categories at the population level, this study selected representative genes with the highest coefficients of variation (CVs) from each category based on the classification results shown in **Supplementary Figure 1B** for detailed analysis (**Supplementary Figure 1B**). The results revealed that genes in the Housekeeping category (e.g., genes from orthogroups OG0000019, OG0000007, and OG0000013) exhibited highly stable expression across the maize inbred lines, with minimal fluctuations between tissues and generally low CV values (<1.5). Genes in the Tissue-biased category showed pronounced expression preferences in specific tissues; for example, OG0000168 displayed relatively high expression in inbred lines such as B73 and II14H, while expression was markedly reduced in Mo17. As the tissue-specificity index (τ) increased, expression divergence at the population level became more pronounced, with Highly specific and Ultra-specific genes showing the strongest differentiation. For instance, in the shoot tissue, OG0000210 was highly expressed in inbred lines such as Tzi8 and P39 but was nearly undetectable in NC350. Similarly, OG0000060 exhibited elevated expression in Tzi8 and NC350 in tassel tissue but was substantially reduced in Ms71. Overall, increasing expression divergence among inbred lines was accompanied by stronger tissue specificity.

#### 3.6.2 Expression patterns of paralogous genes within multi-copy orthogroups

The aforementioned analyses revealed significant expression differences of *ZmCYP450* genes across different tissues and maize varieties, but the expression consistency among paralogous genes within the same genome remained unclear. To address this question, using B73 as a representative, we systematically analyzed the expression patterns of multi−member ZmCYP450 orthogroups at the individual orthogroup level. The results showed widespread expression divergence within the same orthogroup (**Supplementary Table 10**). Taking OG0000000 as an example, this orthogroup contains three *ZmCYP450* genes. Among them, Zm00001eb339130 and Zm00001eb339150 are predominantly expressed in vegetative tissues such as leaf and root, but their expression levels in root and tassel already show marked differences. In contrast, Zm00001eb339170 exhibits strong tissue specificity, being highly expressed only in the tassel (TPM = 51) while remaining almost undetectable in leaf, root, and shoot. OG0000007 displays a similar pattern: Zm00001eb097920 is mainly expressed in leaf (TPM = 9), whereas Zm00001eb100210 is significantly enriched in root and shoot (TPM = 54 and 31, respectively), and Zm00001eb100200 is predominantly expressed in shoot (TPM = 21). Overall, paralogous genes within a subset of multi−copy orthogroups exhibit clear tissue expression differentiation characteristics.

## 4 Discussion

The cytochrome P450 superfamily is the largest enzyme family that plays a key role in plant evolutionary metabolic diversification (Nelson and Werck‐Reichhart, 2011). Members of this family play a catalytic role in a variety of biochemical reactions, and they can produce a variety of secondary metabolites including flavonoids, anthocyanins, isoflavones and terpenoids. As more and more genome-wide sequences are mined, *CYP450* gene superfamily members have been systematically identified and analyzed in a variety of plants, such as tea, sweet potato, wheat and so on. In previous studies, it is not uncommon to perform genome-wide analysis of the CYP450 family for a single reference genome (Zhang et al., 2023c; Sha et al., 2026). However, such studies are often based on specific genetic backgrounds, and it is difficult to fully reveal the structural variation and evolutionary dynamics of the superfamily at the intraspecific level. There is a lack of systematic analysis of gene expansion, deletion and functional differentiation. As an important gramineous crop (Jiang and Liu, 2023), maize has a complex genetic background and significant differences among varieties. It is an ideal material for studying plant metabolic diversity and gene family evolution. In recent years, the development of pan-genomics has provided a new perspective for the analysis of intraspecific genetic variation. However, there is still a lack of systematic pan-genomic research on the ZmCYP450 superfamily. Therefore, the systematic comparison of cross-maize varieties using pan-genomic analysis is helpful to better understand the evolutionary dynamics and functional diversity of the *ZmCYP450* gene family.

### 4.1 Genomic dynamics and adaptation of the ZmCYP450 family from a pan-genomic perspective

In this study, we identified a total of 282 ZmCYP450 OGs, with most orthogroups maintaining 1–5 copies across different varieties, indicating that the overall gene copy number of this family is relatively stable (**Figure 1A**). However, pan-genomic accumulation curves indicate that as the number of included varieties increases, the cumulative number of detected OGs continues to rise, exhibiting a typical open pan-genome trend (Tettelin et al., 2005); simultaneously, the number of core OGs gradually decreases with increasing variety count, reflecting extensive variation in genomic content among individuals within the same species (Hirsch et al., 2014) (**Figure 1B**). Given the critical role of CYP450 enzymes in hormone synthesis and secondary metabolism, this trend is likely related to maize’s adaptation to different environments (Gao et al., 2019); that is, different varieties regulate their metabolic capacity to some extent by retaining or losing certain core OGs to cope with diverse growth conditions. Therefore, relying solely on core OGs is insufficient to fully elucidate intraspecific genetic variation; it is necessary to also focus on genomic regions exhibiting specific distributions among different individuals (Li et al., 2014). Within the ZmCYP450 family, core OGs account for 63.8%, indicating that most ZmCYP450 orthogroups perform basic metabolic functions and are stably retained across different varieties, thereby maintaining the stability of fundamental physiological processes. Notably, private OGs were not detected in any of the 27 varieties, and soft-core OGs and dispensable OGs accounted for the remaining proportion (**Figure 1C**). This suggests that during the long process of maize domestication and breeding, extensive gene flow has occurred among different populations within the ZmCYP450 family (Hufford et al., 2013), resulting in no single gene evolving in isolation, but rather relying more on presence/absence variants (PAV) and copy number variations (CNV) across different varieties (Swanson-Wagner et al., 2010). Previous studies have reported that a significant proportion of genes exhibit marked expression differences between different parents and tissues, and display certain tissue-specific expression patterns (Zhuang and Adams, 2007), which is consistent with the findings in this study that transcripts of genes from dispensable OGs were detected in only a limited number of tissues (**Figure 1D**).

Analysis of presence-absence Variation (PAV) and copy number variants (CNV) further revealed significant differences among varieties. In contrast to the conservation of core components, dispensable OGs exhibited extensive gene deletions in most varieties; this complex genomic composition confirms the presence of substantial structural variation in the ZmCYP450 family within the maize genome (Lu et al., 2015). This phenomenon suggests that the specificity of the ZmCYP450 family stems primarily from dynamic changes in gene number rather than alterations in overall genomic structure. Of course, such widespread gene deletions do not imply a decline in genomic function but are more likely the result of genomic restructuring that occurred during maize’s long-term domestication and dispersal (Hufford et al., 2012). As shown in the figure, we found that lines such as CML322, CML333, and M37W did not exhibit deletions in most soft-core OGs, revealing that certain regulatory genes in these varieties were strongly conserved during domestication (**Figure 2A**); these genes may be considered potential targets for plant genetic improvement (Zhang et al., 2023b). Further CNV analysis indicates that approximately 8% of copy number differences across varieties suggest these regions have been subject to distinct selective pressures (**Figure 2B**). Notably, within core OGs, we observed two distinct CNV patterns: most core OGs are strictly maintained at low copy numbers across varieties, while a minority of orthogroups exhibit differential copy number variations. Genes within these highly variable OGs exhibiting divergent copy number variations are enriched for functional associated with variety-specific traits (Mei et al., 2020), which will have a profound impact on maize phenotypes, traits, and the species’ evolutionary trajectory (Dang et al., 2025). Therefore, the structural dynamics of the ZmCYP450 family, encompassing both gene copy number variation and localized expansions, represent a key functional adaptability and functional adaptability in maize (Tan et al., 2017). Certainly, further validation of representative PAV and CNV loci indicated that the variation patterns inferred from orthogroup occupancy indeed reflect genuine genomic structural differences. Large−scale deletions at PAV loci confirmed gene loss in specific varieties (**Figure 3A**), while the extra copies at CNV loci were supported by local microsynteny (**Figure 3B**), suggesting their origin from genuine duplication events. These results independently validated that the observed PAV and CNV signals are authentic and not attributable to annotation or assembly artifacts (Springer et al., 2009).

### 4.2 Duplication, structural variation, and selective pressure collectively shape the evolutionary mechanisms of the ZmCYP450 family

Maize underwent a relatively recent WGD event in its evolutionary history (approximately 5–12 million years ago), which provided abundant genetic material for the expansion of gene families (Schnable et al., 2011). The long regions of synteny observed in our syntenic dot plot can be regarded as important evidence of this ancient polyploidization event (**Figure 4**). The results indicate that the expansion of the ZmCYP450 gene copies was primarily driven by proximal duplication, followed by DSD, WGD, and TD, while TRD accounted for the smallest proportion (**Figure 5B**). This distribution pattern is consistent with the general principles of genome duplication in plants, namely that gene duplication primarily arises from the synergistic effects of WGD events and local duplication events (Panchy et al., 2016). Following WGD, unequal expression between plant genomes leads to the preferential loss of certain genes from one parental subgenome (Freeling et al., 2012). During this process, many homologous gene pairs generated by WGD undergo intrachromosomal recombination due to the loss of repetitive sequences on either side of the gene or the extensive insertion of transposons; this process is termed fractionation, and such non-canonical recombination is the primary mechanism driving fractionation. It should be noted that this mechanism is not deleterious; while genes lose certain segments, they also rearrange into new regulatory patterns, making gene function more flexible (Woodhouse et al., 2010). In the colinearity map, tandem duplication and proximal duplication events are primarily distributed near the diagonal and exhibit localized high-density clusters, indicating that gene family expansion tends to occur in specific regions of the chromosome (**Figure 5A**). These localized duplication events are not random but result from high evolutionary activity in these regions (Cannon et al., 2004). In contrast, homologous genes generated by whole-genome duplication (WGD) and scattered duplication exhibit a more dispersed distribution. Through chromosome comparisons of B73 vs. Mo17 and B73 vs. Oh7B, we observed longer colinear segments, whereas other varieties exhibited a more fragmented colinearity structure. This may be due to the fact that, compared to B73, these two varieties have undergone more recent genomic structural variations. In particular, a comparison of the B73 and Mo17 genomes by Sun et al. revealed that approximately 10% of the genes between the two are non-collinear, and over 20% of the genes harbor large-effect mutations or structural variations (Sun et al., 2018). These specific segments arise independently at non-linked positions within the genome and are subsequently retained (Eichten et al., 2011); therefore, we speculate that the situation in Oh7B is similar. This implies that the specificity and diversity among varieties are largely generated, lost, or retained independently across different varieties through this non-collinear, dispersed mechanism. Comparative collinearity analysis further indicates that the positions of most *ZmCYP450* genes are highly conserved across different varieties; however, subtle variations in gene order are still observed in certain genomic regions. Chromosome 5 is particularly notable, where cross-chromosomal oblique connections were detected (Burnham et al., 1972). These patterns likely reflect subtle local deviations in gene order rather than large-scale structural rearrangements, contributing to intraspecific genomic variation in maize. Such minor variations may subtly influence gene regulation and phenotypic diversity, although most effects are not readily observable at the macroscopic level (Crow et al., 2020).

Analysis of Ka/Ks ratios indicates that the evolution of the ZmCYP450 family exhibits high overall conservation (**Figure 6**); a mean Ka/Ks value below 0.5 is a typical signal of strong purifying selection (Mallikarjuna et al., 2024). This suggests that purifying selection acting on the ZmCYP450 gene family as a whole is a widespread phenomenon rather than an exception in the maize genome (Yan et al., 2024), and this strong purifying selection may have a significant impact on the evolution of ZmCYP450 (Zhang et al., 2023a). However, we also identified specific orthogroups exhibiting signatures of intense positive selection (Ka/Ks > 1), most notably OG0000003 and OG0000008, which yielded Ka/Ks values exceeding 3. OG0000003 corresponds to the CYP85 family (e.g., Zm00020ab072260 and Zm00021ab290340), which comprises several rapidly evolving members. Similarly, OG0000008 is primarily associated with the CYP72 family, encompassing divergent loci such as Zm00026ab160340 and Zm00042ab164570. Given that positive selection sites are often enriched in substrate-binding regions and active sites, this intense selective pressure has likely modified protein structures, thereby conferring novel catalytic functions on these specific genes (Almeida et al., 2016). We speculate that these rapidly evolving CYP85 and CYP72 subfamilies serve as key drivers of maize species-specific responses to environmental challenges. Collectively, these findings reveal that while the ZmCYP450 family preserves basic metabolic stability, it simultaneously expands potential functional diversity through the rapid evolution of specific gene subfamilies to meet distinct developmental and environmental demands.

### 4.3 Evolutionary strategies and functional diversification in the CYP450 family: a balance between conservation and expansion

Phylogenetic analysis indicates that CYP51, CYP710, and CYP711 are among the most conserved CYP450 families in plants; members of these families mostly occur as single copies or in very low copy numbers, belonging to single-family CYP clans (**Figure 7**). Because they encode fundamental and highly conserved enzymes and have been subject to purifying selection over long periods of evolution, this suggests that such genes serve as ‘core anchors’ for maintaining basic life functions. By being subject to strong purifying selection, they ensure the stability of certain critical fundamental biological processes, such as sterol biosynthesis (Nelson and Werck‐Reichhart, 2011). On the other hand, the CYP71 clan, a typical representative of the A-type, has undergone significant expansion (Wang et al., 2025). The vast number of genes generated by the rapid expansion of this branch not only constitutes the main body of the phylogenetic tree but also reflects the metabolic defense system that maize has constructed over the course of its long-term evolution to cope with complex biotic stresses. For example, many members of the CYP71 family have been shown to participate in the biosynthesis of monoterpenes, including CYP71A1—the first identified gene in the CYP450 family—which participates in the hydroxylation of the monoterpenes nerol and geraniol (Hallahan et al., 1992). Notably, the CYP86 and CYP85 clans, which are involved in lipid oxidation and hormone metabolism, exhibit a dispersed expansion pattern. This independent duplication event across subfamilies stands in sharp contrast to the concentrated expansion of the CYP71 clan. This is because these two clans already existed before the divergence of gymnosperms and angiosperms (Troitsky et al., 1991); their ancient origins and dispersed expansion patterns have led to the distribution of their members across different genomic locations, reflecting a more dispersed and independent pattern of copy accumulation (Wang et al., 2023). We speculate that this distribution pattern facilitates modulation of function across different evolutionary lineages, enabling these genes to respond to ABA and abiotic stress and, consequently, exhibit diverse expression profiles in different tissues (Wang et al., 2023), thereby achieving precise regulation of developmental processes and signal transduction. The *ZmCYP450* gene family fully leverages this unique evolutionary architecture to ensure limited expansion while consistently maintaining the integrity of its core functions, thereby constituting a stable framework for the plant metabolic network.

### 4.4 The dual effects of structural variation on the evolution of the ZmCYP450 family: potential loss of canonical function and expression modulation

Traditional studies have primarily focused on the fine-tuning of gene function by single-nucleotide polymorphisms (SNPs) (Gan et al., 2024), whereas the findings of this study suggest that structural variants (SVs) may serve as a key macro-level mechanism driving the evolution of specific lineages within the ZmCYP450 family. We identified five candidate genes with the most pronounced variation (**Figure 8**). Compared to the reference genome B73, these genes exhibited large-scale deletions of varying degrees across other varieties. Such large-scale genomic deletions have been well documented and are typically preserved in pan-genomes because functionally redundant genes can potentially compensate for the loss, yet the deletion often leads to a potential local loss of function of the gene itself (Liu et al., 2012). To investigate the potential impact of structural variants on gene function, we prioritized the two genes most significantly affected by SVs for in-depth analysis (**Figure 7**). In CML52, Zm00001eb003230 underwent a 3,702 bp deletion covering the predicted promoter and coding regions (**Figure 8**), which severely compromised the gene structure. Such large deletion events may disrupt promoter regions and remove cis-regulatory sequences (**Figure 11A**), thereby reducing transcriptional activity. Given that nearly half of the SVs intersecting coding sequences in maize are significantly associated with transcriptomic variance, these variants often lead to a severe reduction or complete loss of expression, potentially rendering the locus a non-functional allele in the affected accessions (Alonge et al., 2020). Correspondingly, at the protein level, its predicted key catalytic domains (I-helix, K-helix, PERF, and heme-binding site) were completely absent. Because disruption of these core catalytic motifs is widely recognized to severely impair enzyme activity (Schuler and Rupasinghe, 2011), it is highly probable that this gene has lost its canonical function in CML52 (**Figure 10B**). In contrast, Zm00001eb056850 exhibits a distinctly different evolutionary pattern: its predicted protein length is essentially consistent with that of the reference variety, and the typical CYP450 domains remain intact (**Figure 10A**). This conservation suggests that functional constraints may have maintained the integrity of the core CYP450 domains during evolution., constraining its core protein structure to ensure the maintenance of essential physiological activities (Liu et al., 2015). Although non-synonymous substitutions were observed in regions such as 240–250 aa, the core CYP450 domains remained intact, suggesting that the overall protein architecture was largely preserved; this plasticity allows certain members to potentially accommodate new substrates without disrupting the overall folding structure and baseline catalytic capacity (Jung et al., 2011). Furthermore, SV-associated sequence changes in the promoter region altered the distribution of several cis-regulatory elements compared with the reference genotype. (**Figure 11B**). Such structural modifications are generally considered mild and are often insufficient to cause complete gene silencing (Shen et al., 2022), suggesting that the structural variants at this locus manifest as evolutionary expression modulation rather than functional disruption. In summary, the case studies presented here demonstrate that structural variants may play a dual role in the evolution of the ZmCYP450 family: large-scale deletions can lead to loss of gene function at specific loci, whereas moderate SV-associated sequence changes may contribute to expression divergence, thereby facilitating the potential functional diversification of this family across different genetic backgrounds.

### 4.5 The mechanism of ZmCYP450 expression remodeling driven by structural polymorphism and dose nonlinear response

Although the overall copy number of this family remains relatively stable across 27 varieties, polymorphic states such as ‘Typical’, ‘Atypical’, and ‘Missing’ are widely present within it and exhibit a distinct distribution pattern (**Figure 12**). The presence and absence of this gene structure are common in maize individuals (Lian et al., 2024), likely resulting from ongoing genomic reshaping during the domestication process, thereby reflecting a parallel pattern of gene gain and loss (Gui et al., 2022). This phenomenon provides an opportunity to study gene interaction incompatibility (Li and Lee, 2023). Expression analysis revealed that the transcriptional levels of the ZmCYP450 family exhibit highly dynamic fluctuations across different varieties and tissues (**Figure 13**). There is no simple positive correlation between overall transcriptional levels and gene copy numbers, as different genes respond significantly differently to changes in copy numbers; some exhibit patterns consistent with ‘dose compensation’, while others are ‘dose-sensitive’ (Zuo et al., 2016), thereby enabling modulation of the expression network of the expression network (**Figure 14**). Notably, although atypical genes are relatively few in number, they maintain detectable transcriptional activity in multiple tissues, with a particularly elevated expression level in tassel. This confirms that physical gene deletion does not necessarily lead to permanent silencing in all tissues; rather, such genes may be reactivated in specific tissue combinations (Li et al., 2021). In the correlation analysis, we found that the basal metabolic tissues shoot, leaf, and tassel—which are expected to be enriched in gene expression—exhibited a negative correlation with gene expression. This may be due to genomic imbalance; such an inverse correlation causes the expression levels of many genes to decrease when the dosage of certain chromosomal regions increases, thereby restoring balance to the entire gene expression network (Birchler and Yang, 2025).

### 4.6 Expression diversification of *ZmCYP450* genes and its implications for functional adaptation

Studies have shown that the τ-value is an extremely effective method for providing accurate results across different datasets by quantifying the tissue-specific expression of genes (Kryuchkova-Mostacci and Robinson-Rechavi, 2017). Therefore, we chose to incorporate the τ-value to conduct a more comprehensive analysis of tissue expression. Within the ZmCYP450 family, τ-values exhibited a right-skewed distribution overall, with a median of 0.71 (**Figure 15**). This high degree of specificity suggests that the diversity of the ZmCYP450 family is specifically designed to fulfill tissue-specific functions (Das et al., 2023), meaning that the family contains a large number of tissue-specific genes to enable different tissues to carry out more refined biochemical reactions. Genes classified as “Ultra−specific” (τ > 0.98) further support the above observation: the expression of this class of genes is strictly tissue−restricted, being significantly upregulated in specific tissues while remaining largely silent in others (**Supplementary Figure 1A**). For example, representative genes of orthogroups OG0000060, OG0000104, and OG0000103, which belong to the CYP704 and CYP71 families, exhibit strong tissue specificity. The OG0000060 representative gene Zm00001eb005300 is predominantly expressed in the tassel, whereas Zm00001eb165620 (OG0000104) and Zm00001eb165580 (OG0000103) are highly expressed in the shoot. This spatial expression divergence suggests that these genes are involved in specialized secondary metabolic pathways and perform highly niche biological functions (Wang et al., 2021; Lüleci and Yılmaz, 2022). Beyond tissue-specific expression differences, our study revealed substantial expression variation of *ZmCYP450* genes across different genetic backgrounds (**Supplementary Figure 1B**). Notably, two orthogroups, OG0000210 and OG0000060, exhibited pronounced expression divergence among maize inbred lines. The genes Zm00001eb404780 (OG0000210) and Zm00001eb005300 (OG0000060) displayed striking differences in expression levels across distinct genetic backgrounds and tissues. This phenomenon suggests that, in addition to tissue-specific regulation, genetic background may also represent an important factor shaping the expression patterns of *ZmCYP450* genes, consistent with previous findings reported in maize CYP450 studies (Xian and Zhang, 2018). Notably, these two orthogroups belong to the Highly specific and Ultra-specific categories, respectively, and their expression divergence was largely confined to a limited number of specific tissues rather than occurring uniformly across all tissues. Previous studies have demonstrated that the same gene can undergo functional differentiation in different genetic backgrounds by modulating its own expression level, thereby altering the expression of downstream responsive pathways and ultimately influencing plant phenotypes (Sun et al., 2024). Therefore, the pronounced expression divergence observed in these highly tissue-specific genes may reflect an important mechanism underlying functional diversification within the maize population.Based on these findings, future studies integrating CRISPR/Cas9-mediated genome editing with phenomics approaches will facilitate a deeper understanding of the biological functions of these highly tissue-specific genes, particularly their roles in secondary metabolite biosynthesis and accumulation, as well as their contributions to biotic and abiotic stress responses.

In addition to expression differences among varieties, the expression divergence of intra−genomic paralogous *ZmCYP450* genes demonstrates that genes within the same genome can establish distinct expression patterns across different tissues. For example, in OG0000000(Zm00001eb339130, Zm00001eb339150, Zm00001eb339170) and OG0000007 (Zm00001eb097920, Zm00001eb100200, Zm00001eb100210), the tissue expression preferences of individual members complement each other, allowing genes from the same orthogroup to differentiate and perform their own unique physiological functions. These expression divergence events may provide a potential basis for putative neofunctionalization among members of the ZmCYP450 family, thereby facilitating the precise regulation of metabolic responses in various organs of maize (Hughes et al., 2014).

## 5 Conclusion

This study conducted a systematic analysis of the ZmCYP450 family at the pan-genomic level, revealing extensive structural variation and intraspecific diversity despite an overall conserved background. The results indicate that this family exhibits both stability and plasticity during evolution, maintaining core functions while continuously generating new functional potential through genetic variation. Building on these findings, this study established an analytical framework integrating structural variation, evolutionary patterns, and expression profiles, thereby deepening our understanding of the functional differentiation and evolutionary diversification of the ZmCYP450 family. These results not only enrich our understanding of the evolutionary mechanisms of the ZmCYP450 family but also provide valuable insights for maize molecular breeding and related functional studies.

## Data Availability

The original contributions presented in the study are included in the article/**Supplementary Material**. Further inquiries can be directed to the corresponding author.
