# Supplementary figures and images for: Pan-genome analysis reveals structural variation- associated expression and evolutionary diversity of the ZmCYP450 gene family in maize
Source: Front Plant Sci. 2026 Jul 15;17:1874015. doi: 10.3389/fpls.2026.1874015 (PMC13430558; doi:10.3389/fpls.2026.1874015)

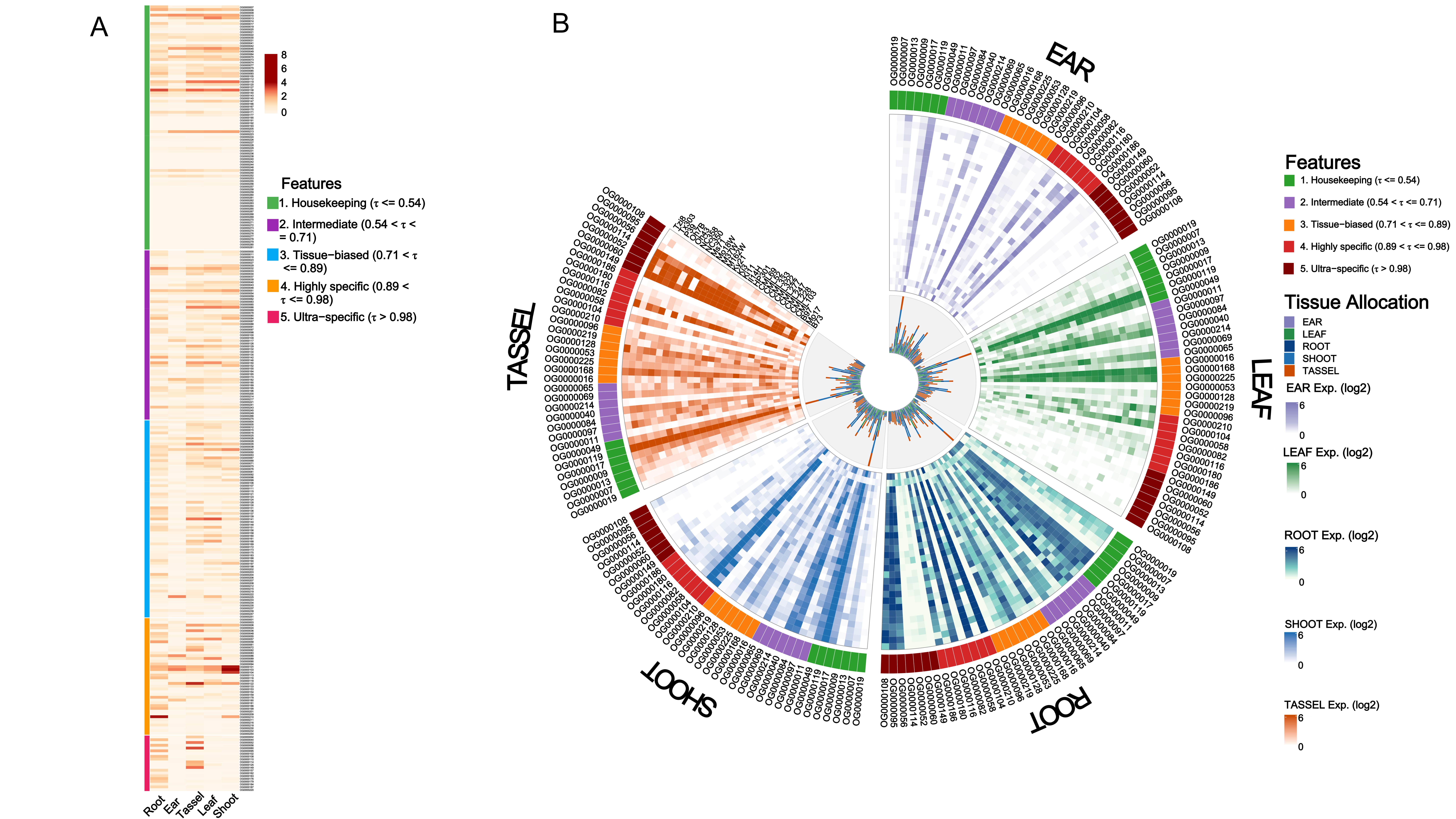

Supplement: Supplementary file 11 [file Image1.jpg]
